# Defining the high-translational readthrough stop codon context

**DOI:** 10.1371/journal.pgen.1011753

**Published:** 2025-06-25

**Authors:** Daniela Smoljanow, Dennis Lebeda, Julia Hofhuis, Sven Thoms

**Affiliations:** Department for Biochemistry and Molecular Medicine, Medical School OWL, Bielefeld University, Bielefeld, Germany; Harvard Medical School, UNITED STATES OF AMERICA

## Abstract

Translational termination is not entirely efficient and competes with elongation, which might result in translational readthrough (TR). TR occurs when a near-cognate tRNA binds to a stop codon, (mis)interpreting it as a sense codon and producing a C-terminal extension of the protein. This process is influenced by the stop codon itself and the surrounding nucleotide sequence, known as the stop codon context (SCC). To investigate the role of these cis-acting elements beyond the high-TR motif UGA CUA G, this study examines specific positions within the SCC, both upstream and downstream of the motif, that contribute to variations in basal and aminoglycoside-induced TR. In particular, we identified a surprisingly large influence of the upstream nucleotide positions -9 and -8 (relative to the stop codon) and positions +11 and +12 on readthrough levels, revealing a complex interplay between nucleotides in the expanded SCC with effects turning out to be non-linear and, furthermore, not transferable to evolutionarily non-adapted SCCs. These findings support our understanding of translational termination and may benefit the development of pharmacological therapy for diseases caused by premature stop codon mutations.

## Author summary

The termination of the production of any protein in the cell is controlled by stop signals (stop codons) on the messenger-RNA (mRNA). A decade ago, we and others discovered a short stretch immediately following the stop codon that renders the termination process leaky so that the stop codon is not read as a stop signal and proteins are extended, creating new proteins (isoforms) with potential additional functions or different locations within the cell. This process is named functional translational readthrough. Lactate and Malate dehydrogenases and the neuronal water channel Aquaporin 4 are known to have isoforms that derive from translational readthrough. The present study examined how specific base positions in the mRNA, before and after the stop codon, influence readthrough. We discovered that specific mRNA bases, both upstream and downstream of the stop codon, play an important and complex role. We also show that the individual elements of these high-readthrough sequences cannot linearly be transferred to disease-related sequences containing pathological premature stop signals. This distinction is important for efforts to develop therapies that aim to overcome such premature stop signals. Our results highlight that strategies promoting therapeutic readthrough must consider the unique sequence features of each stop signal context, rather than relying on general rules derived from high-readthrough signals.

## Introduction

Translation of mRNA is orchestrated by ribosomes and usually terminates when a stop codon enters the ribosomal A site. Eukaryotic cells almost universally recognize the three stop codons UAA, UAG, and UGA. Upon introduction of a stop codon in the ribosomal A site, the eukaryotic release factor 1 (eRF1) binds to the stop codon via its N-terminal domain [1,2]. In eukaryotic cells, eRF1 recognizes all three stop codons, while the class II GTPase eRF3 facilitates termination through GTP hydrolysis, releasing the nascent polypeptide chain [3–5]. Termination is highly accurate, surpassing 99.9% efficiency [6,7]. However, a near-cognate tRNA (nc-tRNA) with two complementary nucleotides can compete with eRF1 for stop codon binding, which may lead to decoding of the stop codon as a sense codon, a process known as translational readthrough (TR). This results in C-terminally extended proteins. This competition is influenced by the availability and properties of tRNAs, including their abundance, modifications, and affinity to specific codons [8–14].

The three stop codons exhibit different termination efficiencies, with UGA showing the lowest termination efficiency and highest TR, followed by UAG and UAA [6,15]. The efficiency of nc-tRNA binding can be influenced by the nucleotide immediately following the UGA stop codon. The presence of a cytosine right after UGA crucially promotes the nc-tRNA binding [11,16,17]. Electron cryo-microscopy structures of mammalian ribosomal complexes have shown the interaction of eRF1 with the + 4 nucleotide immediately following the stop codon, highlighting the importance of this interaction in regulating stop codon recognition and termination efficiency [3,18,19]. In addition, further nucleotides near the stop codon are known to influence the TR efficiency [16,20–23]. Both, the 5’ and 3’ nucleotides of the stop codon context (SCC) contribute significantly to readthrough efficiency and can lead to varying levels of extended protein production [14,24,25]. Data from machine learning analysis of readthrough efficiency from HEK293T ribosome profiling experiments show evidence for a conserved SCC and 3’UTR length that regulate TR [14].

TR can exhibit functional consequences within the cell if the C-terminally extended protein acquires a feature that differs from that of the parental protein, a mechanism known as functional TR (FTR) [26–28]. FTR increases the proteome’s functional capacity without extending the genome. The intricate interplay between precise termination and TR underscores the complexity of translational regulation, allowing the synthesis of diverse protein isoforms from the same mRNA template [10,17,26,27].

FTR was first described for viral genes [20,29,30]. Phylogenetic analysis and ribosome profiling in Drosophila and other metazoa revealed a large number of genes involved in TR [12,31,32]. FTR has also been found in mammals [11,32,33]. Using an in-silico regression model, our group identified a TR-promoting nucleotide context in more than 50 human genes, with experimental validation confirming TR in six of them by measurements in several cell types. The nucleotide consensus sequence CUA G downstream of the UGA stop codon confers very high TR propensity, and deletion experiments showed that the downstream CUA G sequence is a key determinant of TR efficiency [11,17].

Notably, positions outside the high-TR contexts impact TR efficiency. Specific sequences downstream of the stop codon, including the +7, +8, and +9 positions, also determine TR in yeasts, potentially by forming secondary structures [22]. Furthermore, the immediate nucleotides upstream of the stop codon have been shown to modulate TR in bacteria and yeasts [34] and also in mammalian cells. In experiments conducted on mouse fibroblasts (NIH3T3) and HEK293T cell lines, the highest TR levels were observed when the first position upstream of the termination codon was occupied by adenine or guanine, while uracil at this position is associated with lower TR levels [11,35]. In yeast, adenine at positions -1 and -2 enhances TR, particularly for the UAG stop codon, but also for other termination codons [36,37].

The importance of the nucleotide composition surrounding the stop codon, both 3’ and 5’, is underscored by their impact on TR efficiency in various FTR transcripts, such as *Malate dehydrogenase 1* (*MDH1*), *Aquaporin 4 (AQP4)*, and *Lactate dehydrogenase B* (*LDHB*) affecting the C-terminal extension of the expressed proteins [17,28,33]. Due to the presence of the high-TR motif, all these transcripts undergo FTR but exhibit markedly different TR efficiencies. Both TR-extended isoforms, MDH1x and LDHBx (x for extended), gain a peroxisomal targeting signal (PTS1) for import into the peroxisome [17,28,33]. MDH1 is essential for redox balance in the citric acid cycle, and for the malate-aspartate shuttle. In the peroxisome, MDH1x and LDHBx assist in the regeneration of redox equivalents. A recent study showed that MDH1x regulates NAD(H) levels in peroxisomes [38]. The readthrough-extended Aquaporin 4 variant (AQP4x) is specifically localized at astrocytic endfeet surrounding brain blood vessels and modulates the supramolecular organization of AQP4 in astrocytic membranes [39,40].

A comprehensive understanding of the SCC and its impact on TR efficiency is crucial for advancing our knowledge of translational control. The influence of the SCC is investigated in this study by analyzing nucleotide contributions at key positions, focusing on FTR transcripts *MDH1*, *AQP4*, and *LDHB*, which generate well-characterized gene products with regard to the functional consequences of endogenous TR*.* Despite the same high-TR motif in these FTR transcripts, the reason for their different TR levels remains unclear. By interchanging upstream and downstream sequences beyond the high-TR motif and systematically exchanging specific nucleotide positions between *MDH1* and *LDHB*, we detect the influence of the upstream and downstream sequence beyond the high-TR motif.

## Results

### The influence of the 5’ context on translational readthrough efficiency surpasses that of the 3’ context beyond the CUA G motif

**Fig 1:**
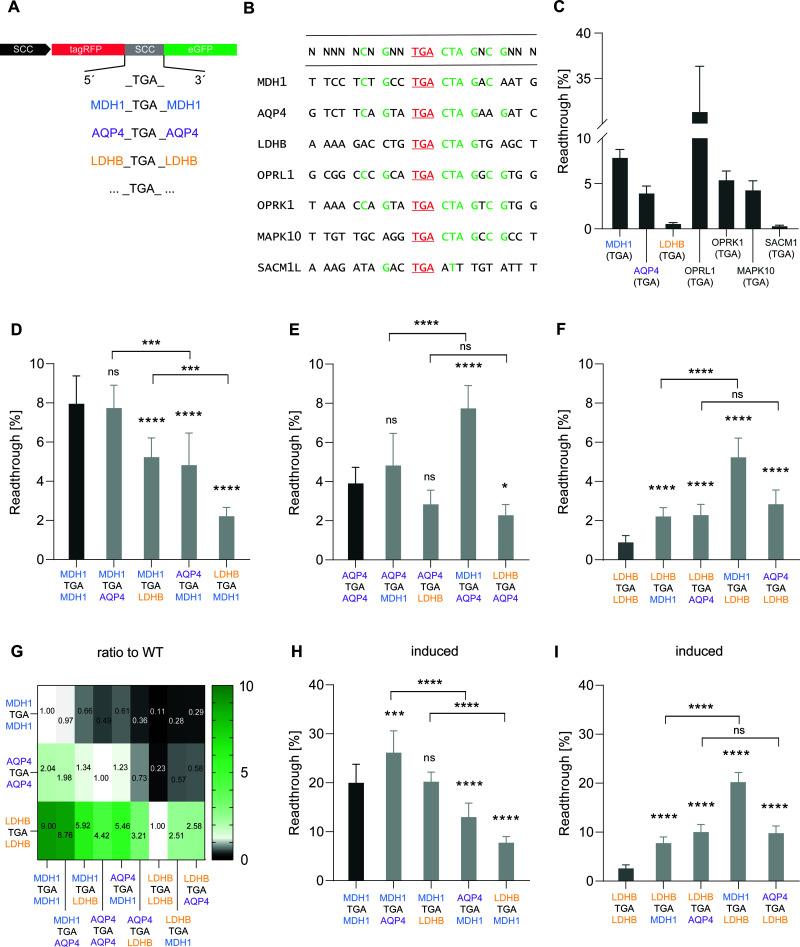
The stop codon context influences both basal and induced translational readthrough. The upstream sequence has the next largest impact beyond the high TR motif. **A)** Schematic: The SCC is located between a 5’ tagRFP and a 3’ eGFP. Upon TR, a RFP-GFP fusion protein is formed. **B)** Alignment of stop codon contexts of TR transcripts, highlighting conserved nucleotide positions. Green letters indicate nucleotides that are present in more than 50% of the sequences at the specific position. **C)** Basal TR of WT SCCs was measured in HeLa cells in three experiments with three replicates (n = 9). **D-F)** Basal TR efficiency of WT SCC sequences of MDH1, AQP4, and LDHB, and cross-combined SCC constructs. Data from at least three experiments and three replicates. **D)** WT sequence of MDH1 with hybrid SCC constructs (MDH1/AQP4 and MDH1/LDHB). **E)** WT sequence of AQP4 with hybrid SCC constructs (AQP4/MDH1 and AQP4/LDHB). **F)** WT sequence of LDHB with hybrid SCC constructs (LDHB/MDH1 and LDHB/AQP4). Values are presented as mean with standard deviation of the mean. Statistical analysis was performed using one-way ANOVA and Dunnett’s multiple comparison test. **G)** Heat Map: Ratios of basal TR levels of WT SCCs (MDH1, AQP4, LDHB) and cross-combined SCC constructs compared to WT reference. **H-I)** Comparison of induced TR in WT MDH1 and LDHB with hybrid SCC constructs (MDH1, AQP4, LDHB). Statistical analysis was performed using one-way ANOVA and Bonferroni’s multiple comparison test.

The transcripts of *MDH1*, *AQP4,* and *LDHB* regulated by FTR share the same high-TR motif but exhibit different levels of readthrough efficiency. To quantify TR, we used a dual reporter assay, ensuring independence from variations in mRNA expression levels [41]. This reporter system encodes an N-terminal tagRFP and a C-terminal eGFP, separated by the SCC, which encompasses nucleotide positions -10 to +13, including the stop codon at positions +1 to +3 (Fig 1A). The system is based on high-content flow cytometry measurements of transiently transfected HeLa cells in 96-well plates [42]. TR is determined as the ratio of eGFP to tagRFP fluorescence, with normalization against a 100% TR control, a construct without a stop codon in the SCC for continuous in-frame translation of eGFP. This construct serves as a reference for maximal eGFP fluorescence. As an independent control, tagRFP fluorescence intensity was used to verify comparable expression of all constructs, ensuring that the observed differences in TR efficiency were not due to variations in plasmid expression levels (S1A Fig).

We first tested the wild-type SCCs of the functionally validated TR transcripts *MDH1, AQP4,* and *LDHB*, all of which naturally terminate with UGA, in HeLa cells to validate differences between these sequences. The results showed a TR efficiency of 7.8% for the *MDH1* SCC. In contrast, the *AQP4* SCC exhibited a lower TR of 3.9%, resulting in a 2-fold difference between *MDH1* and *AQP4*. Notably, the *LDHB* SCC showed the lowest TR level of the functional TR transcripts (Fig 1C) with a > 15-fold difference compared to *MDH1*, and a > 7-fold difference compared to *AQP4*. Despite sharing the UGA stop codon and the CUA G motif known to promote TR, these functionally validated transcripts exhibited markedly different TR levels (Fig 1B and 1C).

To broaden this analysis, we also compared other published TR candidates, like *OPRL1*, *OPRK1*, *MAPK10*, and *SACM1L* [11], all containing the high-TR motif, except for *SACM1L*. Among these gene products, *OPRL1* exhibited the highest TR efficiency with 31.3%, surpassing even *MDH1*. *MAPK10* and *OPRK1* displayed intermediate TR levels (~4 - 5%), while *SACM1L,* lacking the high-TR motif, showed minimal TR efficiency. Sequence analysis of both, functionally validated and candidate FTR transcripts, revealed additional conserved nucleotides beyond the canonical high-TR motif. In particular, a C at position -5, a G at position -3, and a C at position +9 were frequently present in high-efficiency SCCs (Fig 1B). Transcripts that combine these features exhibit remarkably high TR efficiencies (Fig 1C).

To investigate the relative contributions of the upstream (-10 to -1) and the downstream SCC (+4 to +13), we focused on the confirmed FTR transcripts *MDH1, AQP4,* and *LDHB* and generated cross-combined constructs with the upstream sequence of the stop codon from one transcript combined with the downstream sequence from another transcript*.* Compared to wild-type *MDH1* SCC with 7.8% TR, the combined SCC with upstream *MDH1* and downstream *AQP4* sequence showed no significant change in TR, whereas the reverse arrangement led to a significant decrease in TR level to 4.8% (Fig 1D), corresponding to approximately 60% of the wild-type (Fig 1G). Similarly, combining the upstream *MDH1* sequence with the downstream *LDHB* sequence resulted in a significant decrease in TR to 5.2% (~70% of wild-type *MDH1*). Remarkably, the opposite arrangement with upstream *LDHB* and downstream *MDH1* led to a further significant decrease in TR efficiency to 2.2% (Fig 1D), equivalent to ~30% of wild-type (Fig 1G).

The basal TR efficiency of wild-type *AQP4* increased from 3.9% to 7.7% after the replacement of the upstream SCC with *MDH1* (Fig 1E). Compared to the wild-type, the results indicate a ~ 2-fold increase (Fig 1G). On the other hand, the opposite arrangement increased efficiency to 4.8%, representing a non-significant change. However, combining the upstream *LDHB* sequence with *AQP4*’s downstream sequence decreased TR efficiency to 2.3% (Fig 1E). Exchanging the downstream SCC of *AQP4* with *LDHB* did not alter basal TR efficiency compared to *AQP4* wild-type.

For constructs containing parts of the *LDHB* sequence compared to wild-type *LDHB* SCC, all replacements led to increased TR levels. The basal TR efficiency of *LDHB* SCC enlarged from 0.9% to 5.2% (Fig 1F), a ~ 5.9-fold increase when the upstream *MDH1* sequence was combined with the downstream *LDHB* (Fig 1G). The replacement of the downstream sequence from *LDHB* with *MDH1* showed an increase to 2.2%, a ~ 2.5-fold change (Fig 1F and 1G). Fusion of the upstream *AQP4* sequence with the downstream *LDHB* sequence increased efficiency to 2.8%, while the reverse combination increased the TR efficiency to 2.3% (Fig 1F).

In summary, the ‘best’ SCC (*MDH1*) cannot be improved, while the ‘mid’ SCC (*AQP4*) can be improved only by the 5’ *MDH1* SCC, whereas the ‘weaker’ (high-TR) SCC *LDHB* is improved by any exchange, but received the highest increase by the 5’ *MDH1* SCC. Consistent with recent findings, we observed a greater influence of the 5’ sequence on TR efficiency than the 3’ sequence [43] (Fig 1D-G).

Aminoglycosides like geneticin (G418) are known to induce TR by promoting the misreading of stop codons as sense codons [44,45]. By using G418, it can be determined whether SCCs that naturally promote TR are also more responsive to pharmacological induction, helping to distinguish intrinsic sequence effects from drug-induced TR effects. Our findings demonstrate that SCCs exhibiting high basal TR levels also respond effectively to induction by G418. Induction of wild-type *MDH1* SCC led to a TR efficiency of ~20%, a 2.6-fold increase over the basal level. G418 raised the TR efficiency to 26.2% when the downstream *MDH1* sequence was exchanged with the respective *AQP4* sequence. The upstream *MDH1* sequence combined with the downstream *LDHB* sequence showed no significant effect (Fig 1H). Both, the upstream *AQP4* and the upstream *LDHB* sequences, when combined with the downstream *MDH1* resulted in a reduction of the TR level (Fig 1H). For *LDHB* wild-type SCC, G418 induced the TR to 2.5% (Fig 1I). Overall, a value of 20% TR efficiency was measured for the combination of the upstream *MDH1* sequence and the downstream *LDHB* sequence with G418-induction (Fig 1I), which exhibited an 8-fold increase in TR efficiency compared to the wild-type. The fusion of the upstream *AQP4* sequence with the downstream *LDHB* sequence led to a TR efficiency level of 9.8% (Fig 1I).

In summary, altering the upstream sequence from *MDH1* to *AQP4* or *LDHB* in cross-combined constructs consistently has a large impact on TR efficiency for both basal and induced TR, highlighting the upstream SCC’s dominant role.

### Decoding the distinctive impact: Disparate effects of double and single nucleotide exchanges on translational readthrough efficiency

**Fig 2:**
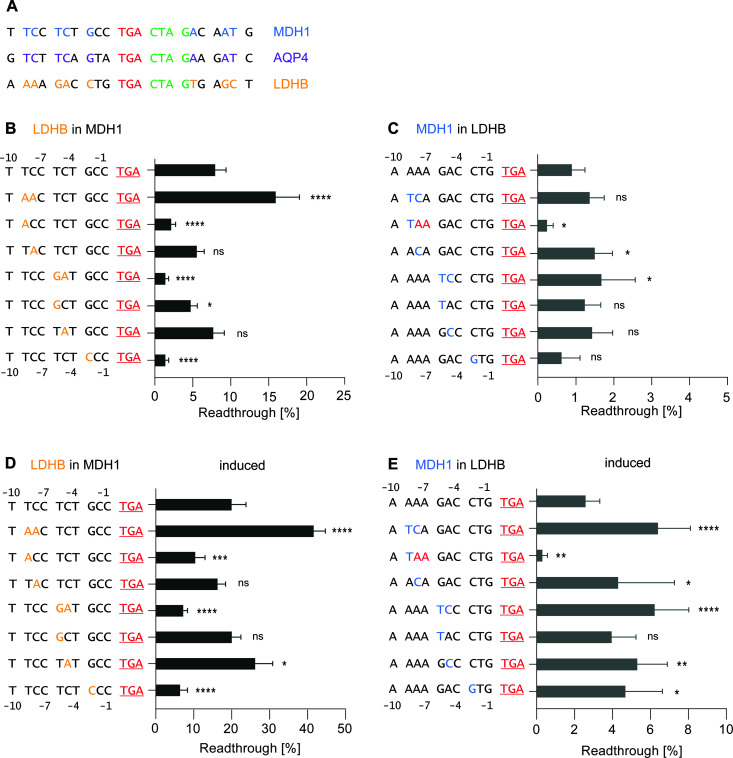
Single and double nucleotide substitutions within the stop codon context lead to changes in translational readthrough efficiency for both basal and inducible readthrough. Single exchanges in the upstream sequence do not exhibit the same tendencies as double exchanges. **A)** SCC of FTR transcripts. Green indicates nucleotides downstream of the stop codon promoting TR (high-TR motif). Blue and purple nucleotides are shared between MDH1 and AQP4. Yellow marks LDHB-specific nucleotides where MDH1 and AQP4 share the same nucleotides. **B-C)** Basal and induced TR of MDH1 SCC with altered upstream positions: **B)** Basal TR of WT MDH1 SCC and constructs with individual nucleotide exchanges. Nucleotides from MDH1 were replaced with corresponding LDHB nucleotides. **C)** Induced TR with G418: Induced TR of WT MDH1 SCC and constructs with individual nucleotide exchanges. **D-E)** Basal and induced TR of LDHB SCC with altered upstream positions: **D)** Basal TR of WT LDHB SCC and constructs with individual nucleotide exchanges. Nucleotides from LDHB were replaced with corresponding MDH1 nucleotides. **E)** G418-induced TR of WT LDHB SCC and constructs with individual nucleotide exchanges. **B-E)** Black letters show the wild-type MDH1 and LDHB sequences. Yellow letters indicate nucleotides from LDHB SCC, and blue letters indicate nucleotides from MDH1 SCC. Statistical analysis was performed using one-way ANOVA and Dunnett’s multiple comparison test.

*MDH1* and *AQP4* showed higher basal TR efficiencies than *LDHB*. *MDH1* and *AQP4* share the high-TR motif (Fig 2A, highlighted in red and green) and further eight SCC nucleotides (Fig 2A, highlighted in blue and purple), which differ in the *LDHB* SCC (Fig 2A, highlighted in yellow). To analyze whether these nucleotides are responsible for the strong effect on TR efficiencies, we systematically exchanged individual nucleotides and nucleotide pairs between *MDH1* and *LDHB* (Fig 2A).

We first investigated the upstream sequence. Surprisingly, an exchange of *MDH1* TC at positions -9 and -8 with *LDHB* AA led to a ~ 2.2-fold increase in TR efficiency from 7.8% to 16% (Figs 2B and S2A). In contrast, the individual exchange at position -9 resulted in a significant decrease in TR (2.2%), while the replacement at position -8 alone showed no effect (Fig 2B). Collectively, the combined exchange at positions -9 and -8 and the single exchanges at position -9 exhibited distinct alterations in the TR efficiency in opposite directions (Figs 2B and S2A). The combined exchange in the *MDH1* SCC at positions -6 and -5 with *LDHB* nucleotides TC → GA decreased TR efficiency to 1.4% (Fig 2B). Similarly, the T → G exchange at position -6 yielded a TR of 4.7%. Replacing the nucleotide G → C at position -3 also significantly decreased TR efficiency to 1.4% (Fig 2B).

Next, we replaced *LDHB* nucleotides within the SCC with *MDH1* nucleotides. The basal TR efficiency of the wild-type *LDHB* SCC was 0.9%. Surprisingly, replacement with *MDH1* nucleotides at positions -9 and -8 (AA → TC) did not alter TR (Fig 2C). In contrast, when we replaced individual nucleotides, switching A → T at -9 decreased apparent TR to 0.3% due to the introduction of an additional in-frame stop codon. The A → C conversion at position -8 increased TR to 1.5% (Fig 2C).

Similarly, the GA → TC exchange at -6 and -5 in *LDHB* increased TR efficiency from 0.9% to 1.7%. The respective individual alterations at these positions did not affect TR significantly (Fig 2C). An exchange at position -3 showed no significant alteration in TR efficiency and exhibited a ratio of ~0.9 (Figs 2C and S2B). Small changes in specific upstream nucleotides can significantly alter TR efficiency, demonstrating that even closely related sequences can have dramatically different effects on TR.

To investigate whether the observed differences in TR efficiency persist under pharmacological induction, the cells were treated with G418. Upon treatment, TR significantly increased for both *MDH1* and *LDHB* sequences. Notably, the TC → AA exchange at positions -9 and -8 in *MDH1* increased TR to 41.6%, equivalent to 2.6-fold compared to non-induced or 2.6-fold compared to wild-type induced, respectively (Figs 2D and S2A). The individual exchange at position -9 in *MDH1* resulted in a higher TR efficiency of 10.4% upon induction but below the induced TR efficiency of the wild-type (Fig 2D).

Surprisingly, for *LDHB* constructs, induction led to TR efficiencies exceeding 6%, a substantial increase from the basal 0.9% (Fig 2E). Specific exchanges demonstrated varying responses to induction. For example, the GA → TC exchange at positions -6 and -5 in *LDHB* resulted in a G418-induced TR efficiency of 6.2%, higher than individual exchanges. This combined exchange exhibited a high ratio to the wild-type, surpassing the G418-induced single exchanges at these positions with ~2.3 compared to the wild-type (Figs 2E and S2B).

Interestingly, combined exchanges of nucleotides located next to each other did not always have the same effect as the corresponding single exchanges within the SCC, as observed for the *MDH1* sequence at positions -9 and -8. It is particularly noteworthy that the varying TR efficiencies appear to result from combinations of nucleotides rather than from individual nucleotides. Specific nucleotide exchanges significantly influenced TR efficiency. Ratios were calculated for G418-induced constructs, revealing consistent behavior across most of them when compared to their non-induced counterparts (S2 Fig). Importantly, while basal TR efficiency varies with nucleotide sequences, G418 induction amplifies these effects in a manner consistent with the basal trends. The nucleotide exchanges that impact TR efficiency at basal levels show similar directional changes under G418 treatment, though with increased magnitude.

### Nucleotides at positions +11 and +12 significantly affect translational readthrough efficiency

**Fig 3:**
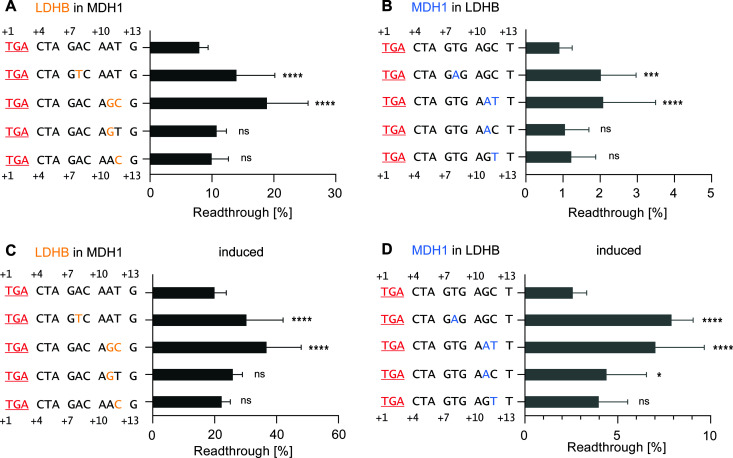
Single and combined neighboring nucleotide exchanges within the downstream SCC affect TR of both, basal and induced readthrough. Single exchanges in the upstream sequence do not display the same patterns as double exchanges. **A-B)** Basal and induced TR of MDH1 SCC with altered downstream positions: **A)** Basal TR of WT MDH1 SCC and constructs with single nucleotide exchanges. Nucleotides from MDH1 were replaced with corresponding LDHB nucleotides. **B)** G418-induced TR of WT MDH1 SCC and constructs with individual nucleotide exchanges. **C-D)** Basal and induced TR of LDHB SCC with altered downstream positions. **C)** Basal TR of WT LDHB SCC and constructs with single nucleotide exchanges. Nucleotides from LDHB were replaced with corresponding MDH1 nucleotides. **D)** G418-induced TR of WT LDHB SCC and constructs with single nucleotide exchanges. **A-D)** Black: WT MDH1 and LDHB sequences. Yellow: Nucleotides from LDHB SCC. Blue: MDH1 SCC nucleotides. Statistical analysis was performed using one-way ANOVA and Dunnett’s multiple comparison test.

To further investigate the influence of downstream nucleotides on TR efficiency, we examined substitutions in the downstream SCC, focusing on *MDH1* and *LDHB*. In the *MDH1* SCC, the A → T substitution at position +8 significantly increased basal TR efficiency to 14% (Fig 3A). Further investigation into *MDH1*’s downstream SCC revealed a more substantial enhancement with the AT→GC exchange at positions +11 and +12. Despite their distance from the stop codon, this alteration boosted TR efficiency to 18.9%, underscoring the profound impact of more distant nucleotides on TR efficiency. Individual substitutions at either +11 (A → G) or +12 (T → C) positions in *MDH1* did not yield significant changes in TR (Fig 3A).

In the *LDHB* SCC, the T → A exchange at position +8 doubled the TR level to 2% (Fig 3B). Similarly, the double exchange at positions +11 and +12 with GC→AT exhibited an increase to 2.1% in TR efficiency, mirroring the effect seen with the + 8-position exchange. Individual substitutions at positions +11 (G → A) and +12 (C → T) in the *LDHB* context showed no significant effect on TR (Fig 3B).

Following induction with G418, various constructs showed significant increases in TR efficiency. Specifically, the exchange at position +8 in *MDH1* with *LDHB* nucleotide led to a TR efficiency of 30.3% (Fig 3C). Exchanging AT→GC at positions +11 and +12 resulted in a substantial TR efficiency increase to 36.8%, significantly higher than induced wild-type *MDH1* SCC. In contrast, individual substitutions at positions +11 and +12 showed no significant alterations compared to wild-type *MDH1* SCC (Fig 3C). When calculating mutant versus wild-type TR ratios, values for basal TR ranged approximately between ~1.2 and ~2.7, whereas the induced ratios ranged between ~1.0 and ~2.3 (S2C Fig).

Examining *LDHB* SCC and corresponding constructs, it was observed that only one construct did not significantly differ from the induced wild-type. The TR value of the wild-type *LDHB* sequence increased from basal 0.9% to 2.6% after induction with G418 (Fig 3D). Induction with G418 and the insertion of adenine instead of thymine at position +8 led to an increase in TR to 7.9% (Fig 3D). The double exchange at positions +11 and +12 (GC→AT) resulted in a TR efficiency of 7%, and the single exchange at position +11 (G → A) led to a measurable G418-induced TR level of 4.4% (Fig 3D). Similarly, the construct with the substitution at position +12 and the T → C exchange yielded a G418-induced TR of 4% (Fig 3D). Mutant versus wild-type TR ratios indicate that the basal TR ratio for the + 8-exchange increased nearly threefold compared to the reference (~2.8). Similarly, the double exchange at positions +11 and +12 exhibited a ~ 2.9-fold increase in TR efficiency compared to the wild-type (S2D Fig).

Exchanges at positions +8, + 11, and +12 reveal that distant nucleotides significantly influence TR efficiency, with double exchanges having pronounced effects. Systematic SCC modifications provide valuable data for in silico TR calculations, enhancing the predictability of FTR candidates and their TR efficiencies. Improved prediction tools can refine the search for FTR candidates.

### Combining nucleotide positions reveals a complex interplay

**Fig 4:**
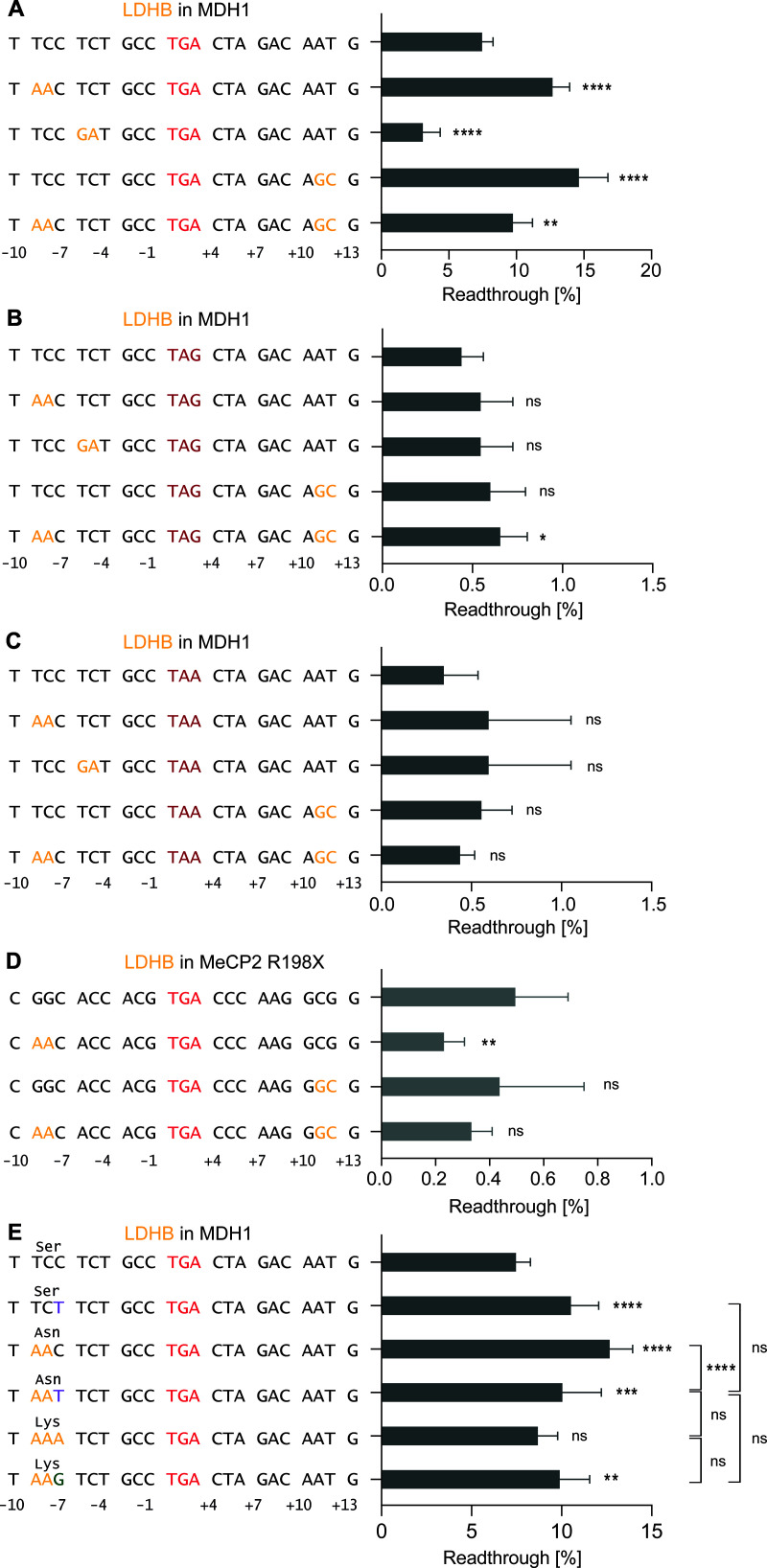
Effect of stop codons, SCC conservation and encoded amino acids. Combined nucleotide substitutions within MDH1 and the TGA SCC alter TR without evidence of additive effects from simultaneous upstream and downstream substitutions of TR-promoting exchanges. **A-C)** Basal TR of WT MDH1 SCC and constructs carrying upstream and downstream, and combined substitutions tested in the context of different stop codons. **A)** TGA stop codon, **B)** TAG stop codon, **C)** TAA stop codon. **D)** Analysis of equivalent substitutions tested in the non-adapted SCC of *MeCP2* R198X. **E)** The amino acid encoded by the nucleotide sequence within the MDH1 SCC at positions -9 to -7 does not account for the observed differences in readthrough efficiency. **A-E)** Black letters: WT MDH1. Yellow letters: Nucleotides from LDHB SCC. **E)** Purple: Nucleotide derived from AQP4. **A-D)** Statistical analysis was performed using one-way ANOVA and Bonferroni’s multiple comparison test. **E)** Statistical analysis was performed using one-way ANOVA and Dunnett’s multiple comparison test.

To ask whether TR can be further enhanced by combining the most potent upstream and downstream exchanges, we generated a construct containing both, the -9 and -8 (TC → AA; 12.7% TR) and the + 11 and +12 (AT→GC; 14.6%) substitutions (Fig 4A). Surprisingly, the combination did not further increase the TR. Instead, the TR dropped to 9.7%, which is lower than either the upstream or the downstream substitutions alone (Fig 4A). These results show that the effects are not additive. Rather, they appear to interfere with or neutralize each other.

To examine whether these effects depend on the stop codon identity, we introduced the most potent nucleotide exchanges into constructs containing either TAG or TAA stop codons. In both cases, neither the upstream (9, -8 TC → AA) nor downstream (+11, + 12 AT → GC) substitutions led to an increase in TR efficiency compared to the respective *MDH1* control (TAG: 0.4%; TAA: 0.3%) (Fig 4B and 4C). Only in the TAG context, the simultaneous exchange of both upstream and downstream nucleotides resulted in a minor increase in TR to a basal level of 0.7% (Fig 4B and 4C).

To explore if the observed SCC effects are transferable to stop contexts that are not adapted in evolution, we investigated the nonsense mutation leading to expression of the truncated p.R198X MeCP2. The premature termination codon (PTC) is associated with Rett syndrome, a neuro-genetic disorder associated with developmental symptoms including learning, language and motor impairment, growth retardation, and seizures [42]. The mutation results in a UGA stop codon followed by a +4 cytosine, but the surrounding sequence does not appear to be optimized for TR. We introduced the nucleotide substitutions that previously showed strong increasing effects in the *MDH1* context into the *MeCP2* nonsense SCC. However, these nucleotides did not enhance readthrough. On the contrary, TR was further decreased from 0.5% in the unmodified *MeCP2* SCC to 0.2% for exchanges at positions -9 and -8 (Fig 4D). We conclude that, at least in this case, TR-promoting mutations in the high-TR context are not transferable to other contexts.

Finally, we assessed whether the observed effects of positions -9 and -8 on TR are primarily driven by the nucleotide sequence or by the identity of the amino acid (encoded by positions -9 to -7) in the nascent polypeptide within the exit tunnel. We combined systematic exchanges at nucleotide position -7 with the previously tested -9 and -8 substitutions. As a control, we implemented a C-to-T mutation at position -7 within the *MDH1* SCC wild-type that does not alter the encoded amino acid (serine). This design allowed us to assess the effect of the nucleotide change alone, without any influence from the altered amino acid. Notably, the single nucleotide substitution showed an increased TR efficiency of 10.5% compared to the *MDH1* wild-type sequence (7.5%) (Fig 4E). To further dissect a possible contribution of amino acid identity, we introduced triple substitutions at positions -9, -8, and -7 encoding either asparagine (AAT, synonymous with AAC) or lysine (AAA, AAG). Surprisingly, both codons for asparagine led to increased TR efficiency, but differed from each other: AAC 12.7%, and AAT 10.0% TR (Fig 4E). In contrast, the lysine-encoding AAA codon (8.7%) did not enhance readthrough compared to wild-type, although encoding the same amino acid as AAG (9.9%), which increased TR (Fig 4E). However, the two lysine-encoding sequences were not associated with significantly different TR levels (Fig 4E). Together, these data suggest that, within a high-TR context, changes at positions -9 to -7 can modulate TR efficiency, even when the encoded amino acid remains the same.

## Discussion

In this study, we show the critical impact of distinct positions within the SCC on TR efficiency, highlighting the intricate interplay of nucleotide combinations within the sequence. Furthermore, the results emphasize the potential combinatorial effects of nucleotides on TR levels.

### The upstream stop codon context has the next largest impact after the high TR motif

To better understand the impact of the 5’ and 3’ SCCs on TR, wild-type SCCs of *MDH1*, *AQP4*, and *LDHB* were tested in HeLa cells, and the effects of the 5’ and 3’ SCCs were systematically investigated by flow cytometry [42]. Combined constructs derived from *MDH1*, *AQP4*, or *LDHB* wild-type demonstrated that the 5’ SCC exerts a stronger influence on the TR efficiency in these SCCs than the 3’ SCC. We observed significant differences in both basal and induced TR efficiencies across 5’ SCC replacements compared to the corresponding wild-type SCC and some 3’ SCC exchanges.

These findings are consistent with recent findings that the 5’ sequence has a substantial role in regulating TR. The TR levels of *OPRL1* and *DUS4L,* including 18 nucleotides upstream and twelve nucleotides downstream of the stop codon and containing the high-TR motif UGA CUA G have been investigated by systematically swapping the upstream sequences. It was shown that the 5’ context accounts for a four-fold difference in TR efficiency compared to a two-fold difference of the 3’ sequence [43]. In our data, the upstream sequence can be responsible for up to a six-fold difference in TR levels compared to the respective wild-type in a non-induced context, further highlighting the regulatory importance.

### Influence of individual nucleotides in the 5’ stop codon context and the combinatorial effect of nucleotides

Earlier studies have analyzed the specific role of 5’ signals in eukaryotes [34–36,46,47]. Interestingly, the insertion of a cytosine at the *MDH1* position -3 significantly reduced TR. This is reminiscent of findings in a case report of a patient with junctional epidermolysis bullosa, where a cytosine at position -3 acts as a determinant of TR efficiency [48].

Strikingly, substitutions at positions -6 and -5 had a strong and inverse effect in *MDH1* (TC → GA) and *LDHB* (GA → TC). The TR level of *MDH1* significantly decreased, whereas that of *LDHB* increased significantly, highlighting the importance of specific nucleotide positions in TR control, which aligns with our in-silico regression model that shows a positive contribution to TR efficiency for TC at positions -6 and -5 [17]. These findings suggest that TR efficiency is fine-tuned by the specific nucleotide composition at the SCCs.

Previous phylogenetic analysis revealed that positions upstream of the stop codon exhibit an evolutionary sequence conservation in TR candidates. Three of five analyzed TR candidates showed conservation of adenines (AA) at the -9 and -8 positions in the genes *OPRK1, OPRL1,* and *SACM1L* in a phylogenetic analysis of 29 mammalian species [11] as present in wild-type *LDHB*. Notably, introducing this AA motif into the upstream sequence of *MDH1* significantly increases the TR efficiency. Alongside these findings, a single substitution of adenine at position -9 leads to a decrease in TR efficiency.

Furthermore, the lack of additivity between upstream and downstream mutations underscores the complexity of SCC-mediated TR regulation. Combinations of mutations that individually enhance TR do not necessarily show synergistic effects. This suggests that optimal TR requires coordinated interactions across multiple sequence elements rather than isolated sequence hotspots. Therefore, models of TR efficiency must consider the broader sequence architecture and non-linearity, especially when predicting or developing readthrough in therapeutic settings.

While the identity of the stop codon is an important factor for TR, the readthrough-promoting differences observed in the TGA context, at positions -9 and -8, as well as +11 and +12, were not found in the TAG and TAA contexts. Due to the generally lower basal TR of those stop codons, such effects may remain below the assay’s resolution. As a result, potential increases may fall within the limited dynamic range of the measurements.

Importantly, these effects are highly context-dependent. Nucleotides that enhance readthrough in evolutionarily adapted SCCs, such as *MDH1*, do not necessarily exert the same effect when transferred into non-adapted contexts like the disease-associated MeCP2 R198X variant, even though this mutation leads to a TGA stop codon followed by cytosine. These results suggest that highly expressed FTR transcripts may have undergone evolutionary selection to optimize TR through their specific SCC composition. With this, evolution does not appear to maximize TR efficiency per se, but rather to tune it to a level that preserves biological function while avoiding the deleterious effects of excessive readthrough.

Our study extends previous findings emphasizing a significant role of nucleotides in the P-site during translational termination (-3 to -1), indicating that variations in the 5’-sequence can be critical determinants of translation termination efficiency [43]. With this study, we redefine the high-TR motif and identify additional nucleotides conserved in high-TR contexts.

### Codon context and sequence architecture as determinants of readthrough efficiency

The translational decoding process selects the appropriate tRNA for an A-site codon with remarkable precision, maintaining error rates between 10^-3^ to 10^-4^. Despite this precision, ribosomes maintain a fast decoding pace of 5 - 20 amino acids per second. This efficiency relies on rapid tRNA screening and engagement with the ribosome [49].

It has previously been proposed that properties of the nascent peptide, such as charge or hydrophobicity, can modulate translation dynamics via interactions with the ribosomal exit tunnel [50,51]. For instance, amino acids like proline are known to significantly slow down peptide bond formation due to steric hindrance and restricted conformational flexibility, which may alter elongation kinetics [50,52]. This has raised the hypothesis that slower incorporation of certain residues could indirectly promote TR by affecting ribosome pausing and termination efficiency.

However, our data indicate that, at least in high-TR contexts of *MDH1*, the nucleotide composition plays a decisive role. This conclusion arises from experiments at positions -9 to -7, where different synonymous codons encoding the same amino acid produce different TR outcomes. Conversely, constructs that encoded different amino acids showed no consistent change in TR efficiency. These findings suggest that codon identity, rather than the translated residue, is the primary driver of TR in this setting. This interpretation aligns with previous findings in mouse cells showing that the codon preceding the stop codon had no amino-acid-dependent effect on TR [35]. While that study focused on the influence of the final incorporated amino acid, our results extend this by demonstrating that the amino acid encoded at codon position -3 does not determine TR.

As the nucleotides -9 to -7 are not present within the ribosome when the stop codon is in the A-site, their effect is unlikely to arise from direct codon-anticodon interactions. Instead, they may influence TR indirectly, for instance, by altering local mRNA structure or modulating the kinetics of elongation prior to termination. Codon-specific elongation rates, shaped by tRNA abundance and codon usage, could affect ribosome speed upstream of the stop codon and thereby influence termination efficiency. Although the precise mechanism remains to be elucidated, our results support a model in which the upstream mRNA sequence contributes to termination efficiency via context-dependent structural and/or kinetic effects.

### Finetuning of the high-readthrough motif influences both basal and inducible translational readthrough efficiency

Translational readthrough-inducing drugs (TRIDs) are being researched for their potential to bypass premature termination codons and recover full-length proteins. Geneticin (G418) is a known standard for experimental TR induction as it provides the properties that allow the ribosome to keep translating but disturb its proofreading function [53–55].

Our findings demonstrate that elevated basal TR levels in chimeric constructs comprising two transcripts correlate with enhanced inducible readthrough. Furthermore, both combined and single-nucleotide exchanges that elevate basal TR levels show improved inducibility of TR with G418 and vice versa, which is particularly relevant for TR of PTCs. These findings suggest that TR therapy is more likely to succeed in the presence of sequence elements showing characteristics of high-TR motifs.

In summary, our study demonstrates that the 5’ and 3’ sequences surrounding the stop codon are crucial for TR efficiency. The complex interplay of these sequences suggests that predicting TR rates requires a detailed understanding of the nucleotide context and further experimental evidence. Additionally, our findings highlight the importance of both upstream and downstream sequences in modulating TR efficiency, with potential implications for therapeutic strategies targeting TR.

## Materials and methods

### DNA constructs

Oligonucleotides containing sequences complementary to the corresponding SCC, with 10 nucleotides upstream and downstream of the stop codon, and overhangs matching the BspEI and BstEII restriction sites were designed. Dual reporter plasmids contained different SCCs and were constructed by annealing (1 µM each) oligonucleotides (Sigma) OST3230 - 3249 and OST3506 - 3541 (S1 and S2 Tables) in annealing buffer (1 mM EDTA in diethylpyrocarbonate-treated H_2_O, 100 mM NaCl, 10 mM Tris/HCl, pH 7.5) in a thermocycler. Oligonucleotides were denatured at 98°C for 5 seconds, annealed at 40°C for 5 seconds, and then kept at 10°C. Annealed oligonucleotides were inserted into BspEI and BstEII sites of pcDNA3.1(+)RFP_MCS_GFP (PST1596) [41].

### Cell culture

HeLa cells were cultured in high-glucose Dulbecco’s Modified Eagle Medium essential medium (DMEM, Gibco) supplemented with 10% heat-inactivated fetal calf serum (v/v) (Biowest), 1% L-glutamine (w/v), 100 µg/mL penicillin/streptomycin (P/S) at 37°C, 5% CO_2_, and 90% humidity. 30,000 cells/well were seeded in 96-well plates for the dual reporter assay.

Cells were transfected with 150 ng of plasmid DNA using Effectene (Qiagen) following the manufacturer’s instructions. The transfection reagent was removed 16 hours after transfection. When indicated, geneticin (Carl Roth) was added at 100 µg/mL for 24 hours.

### Flow cytometry

The cells were cultured in a 96-well plate and prepared for flow cytometry as previously described [41]. Briefly, the medium was discarded, and the transfected cells were washed with 150 µL PBS. Cells were then trypsinized with 35 µL 0.5% trypsin and incubated for 10 minutes at 37°C and resuspended in 165 µL DMEM. Cells were pelleted for 5 minutes at 500 x g (Megafuge 8 centrifuge, ThermoFisher Scientific). The medium was replaced by 200 µL phenol red-free DMEM before flow cytometry (Guava easyCyte, Cytec). Cells were gated by forward scatter between 20,000 and 75,000. Side scatter was set between 7,000 and 60,000. Additionally, the cell population was gated for fluorescence signals (488 nm and 561 nm lasers) based on generating a two-parameter dot-plot of tagRFP detection vs. eGFP detection. The following thresholds were applied: RFP > 700 and GFP > 100. Up to 10,000 gated events were recorded over a 180 s interval per sample. For each biological replicate, three technical replicates were measured for each condition. For each measurement, a 100% TR control (cells transfected with PST1596) was measured in three replicates. Measurements were conducted at least three times with three technical replicates. Data was saved as a flow cytometry standard file (.fcs) for further analysis. To evaluate TR efficiency, the ratio of eGFP to tagRFP fluorescence was normalized to the 100% TR control (PST1596). All gating and calculation steps were performed in RStudio utilizing the R programming environment.

### Statistics

Statistical analysis was done with Prism 10 (Graph Pad) using the one-way ANOVA. When comparing each column with every other column, Bonferroni’s test for multiple comparisons was applied. Dunnett’s test for multiple comparisons was used when all columns were compared to a single control column. Data are presented as means ± SD (standard deviation of the mean).

## Supporting information

S1 Fig Mean RFP intensity values of constructs used in this study. Each data point represents an individual measurement, bars indicate mean and standard deviation (SD).
(EPS)

S2 Fig Ratios of basal and G418-induced TR. **A-D)** Heat map of TR ratios of WT SCCs and constructs with nucleotide exchanges, relative to reference values. **A)** MDH1 basal and induced TR for upstream sequences. **B)** LDHB basal and induced TR for upstream sequences. **C)** MDH1 basal and induced TR for downstream sequences. **D)** LDHB basal and induced TR for downstream sequences.
(EPS)

S1 Table Plasmids used in this study. (DOCX)

S2 Table Oligonucleotides used in this study. (DOCX)

S1 Data Raw data from all measurements. (XLSX)
