# Supplementary material for: Defining the high-translational readthrough stop codon context
Source: PLoS Genet. 2025 Jun 25;21(6):e1011753. doi: 10.1371/journal.pgen.1011753 (PMC12233894; doi:10.1371/journal.pgen.1011753)
Supplement: S1 Table — (DOCX) [file pgen.1011753.s003.docx]

*S1 Table:* Plasmids used in this study.

| PST | Name | Description |
| --- | --- | --- |
| 1596 | pcDNA3.1- tagRFP MCS eGFP | Dual reporter vector |
| 3125 | 1596-MDH1_-9,-8_AA | MDH1 -10 to +13 SCC in PST1596 positions -9TA,-8CA |
| 3190 | 1596-MDH1_-9_A | MDH1 -10 to +13 SCC in PST1596 position -9TA |
| 3191 | 1596-MDH1_-8_A | MDH1 -10 to +13 SCC in PST1596 position -8CA |
| 3100 | 1596-MDH1_-6,-5_GA | MDH1 -10 to +13 SCC in PST1596 positions -6TG,-5CA |
| 3192 | 1596-MDH1_-6_G | MDH1 -10 to +13 SCC in PST1596 position -6TG |
| 3193 | 1596-MDH1_-5_A | MDH1 -10 to +13 SCC in PST1596 position -5CA |
| 3101 | 1596-MDH1_-3_C | MDH1 -10 to +13 SCC in PST1596 position -3 GC |
| 3102 | 1596-MDH1_+8_T | MDH1 -10 to +13 SCC in PST1596 position +8AT |
| 3103 | 1596-MDH1_+11,+12_GC | MDH1 -10 to +13 SCC in PST1596 positions +11AG,+12TC |
| 3194 | 1596-MDH1_+11_G | MDH1 -10 to +13 SCC in PST1596 position +11AG |
| 3195 | 1596-MDH1_+12_C | MDH1 -10 to +13 SCC in PST1596 position +12TC |
| 3111 | 1596-LDHB_-9,-8_TC | LDHB -10 to +13 SCC in PST1596 positions -9AT,-8AC |
| 3196 | 1596-LDHB_-9_T | LDHB -10 to +13 SCC in PST1596 position -9AT |
| 3197 | 1596-LDHB_-8_C | LDHB -10 to +13 SCC in PST1596 position -8AC |
| 3112 | 1596-LDHB_-6,-5_TC | LDHB -10 to +13 SCC in PST1596 positions -6GT ,-5AC |
| 3198 | 1596-LDHB_-6_T | LDHB -10 to +13 SCC in PST1596 position -6GT |
| 3199 | 1596-LDHB_-5_C | LDHB -10 to +13 SCC in PST1596 position -5AC |
| 3209 | 1596-LDHB_-3_G | LDHB -10 to +13 SCC in PST1596 position -3CG |
| 3113 | 1596-LDHB_+8_A | LDHB -10 to +13 SCC in PST1596 position +8TA |
| 3114 | 1596-LDHB_+11,+12_AT | LDHB -10 to +13 SCC in PST1596 positions +11GA,+12CT |
| 3200 | 1596-LDHB_+11_A | LDHB -10 to +13 SCC in PST1596 position +11GA |
| 3201 | 1596-LDHB_+12_T | LDHB -10 to +13 SCC in PST1596 position +12CT |
| 3185 | 1596-MDH1_TGA_AQP4 | MDH1 -10 to -1, TGA stop, AQP4 SCC +4 to +13 |
| 3184 | 1596-MDH1_TGA_LDHB | MDH1 -10 to -1, TGA stop, LDHB SCC +4 to +13 |
| 3188 | 1596-AQP4_TGA_MDH1 | AQP4 -10 to -1, TGA stop, MDH1 SCC +4 to +13 |
| 3189 | 1596-AQP4_TGA_LDHB | AQP4 -10 to -1, TGA stop, LDHB SCC +4 to +13 |
| 3186 | 1596-LDHB_TGA_MDH1 | LDHB -10 to -1, TGA stop, MDH1 SCC +4 to +13 |
| 3187 | 1596-LDHB_TGA_AQP4 | LDHB -10 to -1, TGA stop, AQP4 SCC +4 to +13 |
| 3481 | 1596-MDH1-9,-8,-7AAT-Ser | MDH1 -10 to +13 SCC in PST1596 positions -9TA,-8CA,-7CT |
| 3482 | 1596-MDH1-9,-8,-7AAA(LDHB)-Lys | MDH1 -10 to +13 SCC in PST1596 positions -9TA,-8CA,-7CA |
| 3483 | 1596-MDH1-9-8,-7AAG-Lys | MDH1 -10 to +13 SCC in PST1596 positions -9TA,-8CA,-7CG |
| 3484 | 1596-MDH1-9,-8exLDHB_AA_TAG | MDH1 -10 to +13 SCC in PST1596 pos. -9TA,-8CA (TGA 🡪 TAG) |
| 3485 | 1596-MDH1_-9,-8 exLDHB_AA_TAA | MDH1 -10 to +13 SCC in PST1596 pos. -9TA,-8CA (TGA 🡪 TAA) |
| 3486 | 1596-MDH1_-6,-5 exLDHB_GA_TAG | MDH1 -10 to +13 SCC in PST1596 pos. -6TG,-5CA (TGA 🡪 TAG) |
| 3487 | 1596-MDH1_-6,-5 exLDHB_GA_TAA | MDH1 -10 to +13 SCC in PST1596 pos. -6TG,-5CA (TGA 🡪 TAA) |
| 3488 | 1596-MDH1_+11, +12 exLDHB_GC_TAG | MDH1 -10 to +13 SCC in PST1596 pos. +11AG,+12TC (TGA 🡪 TAG) |
| 3489 | 1596-MDH1_+11,+12 exLDHB_GC_TAA | MDH1 -10 to +13 SCC in PST1596 pos. +11AG,+12TC (TGA 🡪 TAA) |
| 3490 | 1596-MDH1_-9,-8,+11,+12 exLDHB_TGA | MDH1 -10 to +13 SCC in PST1596 pos. -9TA,-8CA & pos. +11AG,+12TC |
| 3491 | 1596-MDH1_-9,-8,+11,+12 exLDHB_TAG | MDH1 -10 to +13 SCC in PST1596 positions -9TA,-8CA (TGA 🡪 TAG) and positions +11AG,+12TC |
| 3492 | 1596-MDH1_-9,-8,+11,+12 exLDHB_TAA | MDH1 -10 to +13 SCC in PST1596 positions -9TA,-8CA (TGA 🡪 TAA) and positions +11AG,+12TC |
| 3493 | 1596-MDH1_-7T-Ser_SCC | MDH1 -10 to +13 SCC in PST1596 positions -7CT |
| 3494 | 1596-MDH1_WT_TAG | MDH1 -10 to +13 SCC in PST1596 (TGA 🡪 TAG) |
| 3495 | 1596-MDH1_WT_TAA | MDH1 -10 to +13 SCC in PST1596 (TGA 🡪 TAA) |
| 3496 | 1596-OPRK1_SCC | OPRK1 -10 to +13 SCC in PST1596 |
| 3497 | 1596-OPRL1_SCC | OPRL1 -10 to +13 SCC in PST1596 |
| 3498 | 1596-MAPK10_SCC | MAPK10 -10 to +13 SCC in PST1596 |
| 3500 | 1596-SACM1L_SCC | SACM1L -10 to +13 SCC in PST1596 |
| 3501 | 1596-p.R198X_-9,-8 AA (LDHB) | MeCP2 R198X -10 to +13 SCC in PST1596 positions -9GA,-8GA |
| 3502 | 1596-p.R198X_+11,+12GC (MDH1) | MeCP2 R198X -10 to +13 SCC in PST1596 positions +11CG,+12GC |
| 3503 | 1596-p.R198X_-9,-8 AA (LDHB), +11,12GC(MDH1) | MeCP2 R198X -10 to +13 SCC in PST1596 positions -9GA,-8GA and +11CG,+12GC |
