# Supplementary material for: Defining the high-translational readthrough stop codon context
Source: PLoS Genet. 2025 Jun 25;21(6):e1011753. doi: 10.1371/journal.pgen.1011753 (PMC12233894; doi:10.1371/journal.pgen.1011753)
Supplement: S2 Table — (DOCX) [file pgen.1011753.s004.docx]

*S2 Table:* Oligonucleotides used in this study.

| OST | Description | Sequence 5´→ 3´ |
| --- | --- | --- |
| 3230 | 3230-MDH1_-9,-8_AA-f | GTCACCG T AAC TCT GCC TGA CTA GAC AAT G T |
| 3231 | 3231-MDH1_-9,-8_AA-r | CCGGA C ATT GTC TAG TCA GGC AGA GTT A CG |
| 3232 | 3232-MDH1_-6,-5_GA-f | GTCACCG T TCC GAT GCC TGA STA GAC AAT G T |
| 3233 | 3233-MDH1_-6,-5_GA-r | CCGGA C ATT GTC TAG TCA GGC ATC GGA A CG |
| 3234 | 3234-MDH1_-3_C-f | GTCACCG T TCC TCT CCC TGA CTA GAC AAT G T |
| 3235 | 3235-MDH1_-3_C-r | CCGGA C ATT GTC TAG TCA GGG AGA GGA A CG |
| 3236 | 3236-MDH1_+8_T-f | GTCACCG T TCC TCT GCC TGA CTA GTC AAT G T |
| 3237 | 3237-MDH1_+8_T-r | CCGGA C ATT GAC TAG TCA GGC AGA GGA A CG |
| 3238 | 3238-MDH1_+11,+12_GC-f | GTCACCG T TCC TCT GCC TGA CTA GAC AGC G T |
| 3239 | 3239-MDH1_+11,+12_GC-r | CCGGA C GCT GTC TAG TCA GGC AGA GGA A CG |
| 3240 | 3240-LDHB_-9,-8_TC-f | GTCACCG A TCA GAC CTG TGA CTA GTG AGC T T |
| 3241 | 3241-LDHB_-9,-8_TC-r | CCGGA A GCT CAC TAG TCA CAG GTC TGA T CG |
| 3242 | 3242-LDHB_-6,-5_TC-f | GTCACCG A AAA TCC CTG TGA CTA GTG AGC T T |
| 3243 | 3243-LDHB_-6-5_TC-r | CCGGA A GCT CAC TAG TCA CAG GGA TTT T CG |
| 3244 | 3244-LDHB_-3_G-f | GTCACCG A AAA AGC GTG TGA CTA GTG AGC T T |
| 3245 | 3245-LDHB_-3_G-r | CCGGA A GCT CAC TAG TCA CAG TCT TTT T CG |
| 3246 | 3246-LDHB_+8_A-f | GTCACCG A AAA GAC CTG TGA CTA GAG AGC T T |
| 3247 | 3247-LDHB_+8_A-r | CCGGA A GCT CTC TG TCA CAG GTC TTT T CG |
| 3248 | 3248-LDHB_+11,+12_AT-f | GTCACCG A AAA GAC CTG TGA CTA GTG AAT T T |
| 3249 | 3249-LDHB_+11,+12_AT-r | CCGGA A ATT CAC TAG TCA CAG GTC TTT T CG |
| 3506 | 3506-MDH1_-9_A_f | GTCACCG T ACC TCT GCC TGA CTA GAC AAT G T |
| 3507 | 3507-MDH1_-9_A_r | CCGGA C ATT GTC TAG TCA GGC AGA GGT A CG |
| 3508 | 3508-MDH1_-8_A_ f | GTCACCG T TAC TCT GCC TGA CTA GAC AAT G T |
| 3509 | 3509-MDH1_-8_A_r | CCGGA C ATT GTC TAG TCA GGC AGA GTA A CG |
| 3510 | 3510-MDH1_-6_G_f | GTCACCG T TCC GCT GCC TGA CTA GAC AAT G T |
| 3511 | 3511-MDH1_-6_G_r | CCGGA C ATT GTC TAG TCA GGC AGC GGA A CG |
| 3512 | 3512-MDH1_-5_A_f | GTCACCG T TCC TAT GCC TGA CTA GAC AAT G T |
| 3513 | 3513-MDH1_-5_A_r | CCGGA C ATT GTC TAG TCA GGC ATA GGA A CG |
| 3514 | 3514-MDH1_11_G_f | GTCACCG T TCC TCT GCC TGA CTA GAC AGT G T |
| 3515 | 3515-MDH1_11_G_r | CCGGA C ACT GTC TAG TCA GGC AGA GGA A CG |
| 3516 | 3516-MDH1_12_C_f | GTCACCG T TCC TCT GCC TGA CTA GAC AAC G T |
| 3517 | 3517-MDH1_12_C_r | CCGGA C GTT GTC TAG TCA GGC AGA GGA A CG |
| 3518 | 3518-LDHB_-9_T_f | GTCACCG A TAA GAC CTG TGA CTA GTG AGC T T |
| 3519 | 3519-LDHB_-9_T_r | CCGGA A GCT CAC TAG TCA CAG GTC TTA T CG |
| 3520 | 3520-LDHB_-8_C_f | GTCACCG A ACA GAC CTG TGA CTA GTG AGC T T |
| 3521 | 3521-LDHB_-8_C_r | CCGGA A GCT CAC TAG TCA CAG GTC TGT T CG |
| 3522 | 3522-LDHB_-6_T_f | GTCACCG A AAA TAC CTG TGA CTA GTG AGC T T |
| 3523 | 3523-LDHB_-6_T_r | CCGGA A GCT CAC TAG TCA CAG GTA TTT T CG |
| 3524 | 3524-LDHB_-5_C_f | GTCACCG A AAA GCC CTG TGA CTA GTG AGC T T |
| 3525 | 3525-LDHB_-5_C_r | CCGGA A GCT CAC TAG TCA CAG GGC TTT T CG |
| 3526 | 3526-LDHB_11_A_f | GTCACCG A AAA GAC CTG TGA CTA GTG AAC T T |
| 3527 | 3527-LDHB_11_A_r | CCGGA A GTT CAC TAG TCA CAG GTC TTT T CG |
| 3528 | 3528-LDHB_12_T_f | GTCACCG A AAA GAC CTG TGA CTA GTG AGT T T |
| 3529 | 3529-LDHB_12_T_r | CCGGA A ACT CAC TAG TCA CAG GTC TTT T CG |
| 3530 | 3530-MDH1_TGA_LDHB-f | GTCACCG T TCC TCT GCC TGA CTA GTG AGC T T |
| 3531 | 3531-MDH1_TGA_LDHB-r | CCGGA A GCT CAC TAG TCA GGC AGA GGA A CG |
| 3532 | 3532-MDH1_TGA_AQP4-f | GTCACCG T TCC TCT GCC TGA CTA GAA GAT C T |
| 3533 | 3533-MDH1_TGA_AQP4-r | CCGGA G ATC TTC TAG TCA GGC AGA GGA A CG |
| 3534 | 3534-LDHB_TGA_MDH1-f | GTCACCG A AAA GAC CTG TGA CTA GAC AAT G T |
| 3535 | 3535-LDHB_TGA_MDH1-r | CCGGA C ATT GTC TAG TCA CAG GTC TGA T CG |
| 3536 | 3536-LDHB_TGA_AQ4-f | GTCACCG A AAA GAC CTG TGA CTA GAA GAT C T |
| 3537 | 3537-LDHB_TGA_AQP4-r | CCGGA G ATC TTC TAG TCA CAG GTC TGA T CG |
| 3538 | 3538-AQP4_TGA_MDH1-f | GTCACCG G TCT TCA GTA TGA CTA GAC AAT G T |
| 3539 | 3539-AQP4_TGA_MDH1-r | CCGGA C ATT GTC TAG TCA TAC TGA AGA C CG |
| 3540 | 3540-AQP4_TGA_LDHB-f | GTCACCG G TCT TCA GTA TGA CTA GTG AGC T T |
| 3541 | 3541-AQP4_TGA_LDHB-r | CCGGA A GCT CAC TAG TCA TAC TGA AGA C CG |
| 4176 | 4176-MDH1-9,-8,-7AAT-Ser_for | GTCACCG T AAT TCT GCC TGA CTA GAC AAT G T |
| 4177 | 4177-MDH1-9,-8,-7AAT-Ser_rev | CCGGA C ATT GTC TAG TCA GGC AGA ATT A CG |
| 4178 | 4178-MDH1-9,-8,-7AAA(LDHB)-Lys_for | GTCACCG T AAA TCT GCC TGA CTA GAC AAT G T |
| 4179 | 4179-MDH1-9,-8,-7AAA(LDHB)-Lys_rev | CCGGA C ATT GTC TAG TCA GGC AGA TTT A CG |
| 4180 | 4180-MDH1-9-8,-7AAG-Lys_for | GTCACCG T AAG TCT GCC TGA CTA GAC AAT G T |
| 4181 | 4181-MDH1-9-8,-7AAG-Lys_rev | CCGGA C ATT GTC TAG TCA GGC AGA CTT A CG |
| 4182 | 4182-MDH1-9,-8exLDHB_AA_TAG_for | GTCACCG T AAC TCT GCC TAG CTA GAC AAT G T |
| 4183 | 4183-MDH1-9,-8exLDHB_AA_TAG_rev | CCGGA C ATT GTC TAG CTA GGC AGA GTT A CG |
| 4184 | 4184-MDH1_-9,-8exLDHB_AA_TAA_for | GTCACCG T AAC TCT GCC TAA CTA GAC AAT G T |
| 4185 | 4185-MDH1_-9,-8exLDHB_AA_TAA_rev | CCGGA C ATT GTC TAG TTA GGC AGA GTT A CG |
| 4186 | 4186-MDH1_-6,-5exLDHB_GA_TAG_for | GTCACCG T TCC GAT GCC TAG CTA GAC AAT G T |
| 4187 | 4187-MDH1_-6,-5exLDHB_GA_TAG_rev | CCGGA C ATT GTC TAG CTA GGC ATC GGA A CG |
| 4188 | 4188-MDH1_-6,-5exLDHB_GA_TAA_for | GTCACCG T TCC GAT GCC TAA CTA GAC AAT G T |
| 4189 | 4189-MDH1_-6,-5exLDHB_GA_TAA_rev | CCGGA C ATT GTC TAG TTA GGC ATC GGA A CG |
| 4190 | 4190-MDH1_+11,+12exLDHB_GC_TAG_for | GTCACCG T TCC TCT GCC TAG CTA GAC AGC G T |
| 4191 | 4191-MDH1_+11,+12exLDHB_GC_TAG_rev | CCGGA C GCT GTC TAG CTA GGC AGA GGA A CG |
| 4192 | 4192-MDH1_+11,+12exLDHB_GC_TAA_for | GTCACCG T TCC TCT GCC TAA CTA GAC AGC G T |
| 4193 | 4193-MDH1_+11,+12exLDHB_GC_TAA_rev | CCGGA C GCT GTC TAG TTA GGC AGA GGA A CG |
| 4194 | 4194-MDH1_-9,-8,+11,+12exLDHB_TGA_for | GTCACCG T AAC TCT GCC TGA CTA GAC AGC G T |
| 4195 | 4195-MDH1_-9,-8,+11,+12exLDHB_TGA_rev | CCGGA C GCT GTC TAG TCA GGC AGA GTT A CG |
| 4196 | 4196-MDH1_-9,-8,+11,+12exLDHB_TAG_for | GTCACCG T AAC TCT GCC TAG CTA GAC AGC G T |
| 4197 | 4197- MDH1_-9,-8,+11,+12exLDHB_TAG_rev | CCGGA C GCT GTC TAG CTA GGC AGA GTT A CG |
| 4198 | 4198-MDH1_-9,-8,+11,+12exLDHB_TAA_for | GTCACCG T AAC TCT GCC TAA CTA GAC AGC G T |
| 4199 | 4199-MDH1_-9,-8,+11,+12exLDHB_TAA_rev | CCGGA C GCT GTC TAG TTA GGC AGA GTT A CG |
| 4200 | 4200-MDH1_-7T-Ser_SCC_for | GTCACCG T TCT TCT GCC TGA CTA GAC AAT G T |
| 4201 | 4201-MDH1_-7T-Ser_SCC_rev | CCGGA C ATT GTC TAG TCA GGC AGA AGA A CG |
| 4202 | 4202-MDH1_WT_TAG_for | GTCACCG T TCC TCT GCC TAG CTA GAC AAT G T |
| 4203 | 4203-MDH1_WT_TAG_rev | CCGGA C ATT GTC TAG CTA GGC AGA GGA A CG |
| 3815 | 3815-MDH1_WT_TAA_for | GTCACCG T TCC TCT GCC TAA CTA GAC AAT G T |
| 3816 | 3816-MDH1_WT_TAA_rev | CCGGA C ATT GTC TAG TTA GGC AGA GGA A CG |
| 4204 | 4204-OPRK1_SCC_for | GTCACCG T AAA CCA GTA TGA CTA GTC GTG G T |
| 4205 | 4205-OPRK1_SCC_rev | CCGGA C CAC GAC TAG TCA TAC TGG TTT A CG |
| 4206 | 4206-OPRL1_SCC_for | GTCACCG G CGG CCC GCA TGA CTA GGC GTG G T |
| 4207 | 4207-OPRL1_SCC_rev | CCGGA C CAC GCC TAG TCA TGC GGG CCG C CG |
| 4208 | 4208-MAPK10_SCC_for | GTCACCG T TGT TGC AGG TGA CTA GCC GCC T T |
| 4209 | 4209-MAPK10_SCC_rev | CCGGA A GGC GGC TAG TCA CCT GCA ACA A CG |
| 4212 | 4212-SACM1L_SCC_for | GTCACCG A AAG ATA GAC TGA ATT TGT ATT T T |
| 4213 | 4213-SACM1L_SCC_rev | CCGGA A AAT ACA AAT TCA GTC TAT CTT T CG |
| 4214 | 4214-p.R198X_-9,-8AA(LDHB)_for | GTCACCG C AAC ACC ACG TGA CCC AAG GCG G T |
| 4215 | 4215-p.R198X_-9,-8AA(LDHB)_rev | CCGGA C CGC CTT GGG TCA CGT GGT GTT G CG |
| 4216 | 4216-p.R198X_+11,+12GC (MDH1)_for | GTCACCG C GGC ACC ACG TGA CCC AAG GGC G T |
| 4217 | 4217-p.R198X_+11,+12GC (MDH1)_rev | CCGGA C GCC CTT GGG TCA CGT GGT GCC G CG |
| 4218 | 4218-p.R198X_-9,-8AA(LDHB), +11,12 GC (MDH1)_for | GTCACCG C AAC ACC ACG TGA CCC AAG GGC G T |
| 4219 | 4219-p.R198X_-9,-8AA(LDHB), +11,12 GC (MDH1)_rev | CCGGA C GCC CTT GGG TCA CGT GGT GTT G CG |
