# Supplementary material for: Defining the high-translational readthrough stop codon context
Source: PLoS Genet. 2025 Jun 25;21(6):e1011753. doi: 10.1371/journal.pgen.1011753 (PMC12233894; doi:10.1371/journal.pgen.1011753)
Supplement: Attachment — Submitted filename: 153738_1_rebuttal_3303107_ss1d1r.docx [file pgen.1011753.s006.docx]

**Manuscript number:** #RC-2024-02839

**Corresponding author(s):** Prof. Sven Thoms

*[The “revision plan” should delineate the revisions that authors intend to carry out in response to the points raised by the referees. It also provides the authors with the opportunity to explain their view of the paper and of the referee reports.*

*The document is important for the editors of affiliate journals when they make a first decision on the transferred manuscript. It will also be useful to readers of the reprint and help them to obtain a balanced view of the paper.*

*If you wish to submit a full revision, please use our "*[*Full Revision*](https://reviewcommons.org/templates/full-revision.docx)*" template.* ***It is important to use the appropriate template to clearly inform the editors of your intentions.****]*

## 1. General Statements [optional]

We thank the reviewers for their constructive and helpful comments. Based on these, we have developed a revision plan as detailed below.

## 2. Description of the planned revisions

*Insert here a point-by-point reply that explains what revisions, additional experimentations and analyses are planned to address the points raised by the referees.*

**Reviewer #1 (Evidence, reproducibility and clarity (Required)):**

The rationale for comparing only three sequences is unclear, given that at least seven sequences have been described with the same UGA CUA G motif. It would be more relevant and informative to compare the differences among these seven sequences rather than focusing on just three. Notably it would be helpful to include the alignment of the three selected sequences alongside OPRL1, OPRK1, AQP4, and MAPK10. These genes also carry the same UGA CUA G motif but likely differ in surrounding nucleotides. In my opinion considering all seven sequences is particularly relevant given that OPRL1 reportedly has a readthrough efficiency of 30%, much higher than the 8% reported for MDH1.

**Response:**

The rationale and the primary goal of our study was to focus those gene products that are best character­ized with regard to the functional consequences of endogenous translational readthrough. The three sequences we originally selected (MDH1, AQP4, and LDHB) have high levels of readthrough and well-documented functional readthrough events with established biological roles. So our initial selection was not based on the sheer presence of the UGA_CUA_G (which can be found in > 50 transcripts) motif, but included a well-established functional relevance. We implicitly assumed that readthrough in highly expressed transcripts that have a clear functional consequence such as the peroxisome import for LDHBx and MDH1x have undergone a stronger evolutionary (sequence) selection than those where the high-readthrough motif is present, but expression is low and/or functional consequences of readthrough is less well established. We thank the reviewer for pointing this out and have now more carefully explained the rationale for our selection in the manuscript.

We also agree that expanding the analysis to all seven sequences carrying the UGA_CUA_G motif could provide further insights into sequence-specific differences in translational readthrough efficiency. To address this, we have done sequence alignments including further candidates (*OPRL1*, *OPRK1*, *MAPK10*) and incorporated this analysis as a new figure (Supplementary Figure 2). This alignment highlights conserved and divergent nucleotide positions, allowing us to contextualize our findings within a broader sequence framework.

Furthermore, we have started cloning these additional sequences and are currently incorporating them into our reporter system to evaluate their TR efficiency under our experimental conditions. These results will be included in the revised manuscript as soon as they are available.

Regarding functional extension evidence for the additional candidates, we are currently reviewing the literature to determine whether *OPRL1*, *OPRK1*, *MAPK10* have documented functional protein extensions resulting from readthrough. If such data exist, we will discuss them in the revised manuscript to provide a more comprehensive perspective on readthrough efficiency across different genes. We appreciate this constructive suggestion and believe that incorporating additional sequences will further strengthen our conclusions.

**Reviewer #1 (Evidence, reproducibility and clarity (Required)):**

I am wondering of the results are limited to the UGA CUA G motif or not? It would be valuable to explore if the same observations hold when replacing UGA CUA G with an inefficient context or with another efficient readthrough motif (e.g., CAA UUA).

**Response:**

As explained above, our intention was to analyze the high-readthrough motif, and UAA and UAG are not part thereof. However, we find the question raised by the reviewer interesting and important, because it could extend of findings to a means of modulating more common stop codon contexts.

To address this point, we have planned additional experiments utilizing a disease-associated premature termination codon (PTC) context, specifically the p.R198X mutation from Rett syndrome, as reported by Lebeda et al., 2024. This SCC has been shown to exhibit relatively high TR efficiency but does not re­present an evolutionarily adapted readthrough context. By testing the effects of -9, -8, +11, and +12 in this alternative SCC, we aim to determine whether the identified nucleotide positions influence TR efficiency in a non-canonical TR context. We believe this will provide valuable insights into the more genera­l context of our findings and we thank the reviewer for raising this important point.

The discussion extensively addresses the importance of amino acids in the ribosome exit tunnel. To strengthen this argument, the authors should consider experiments that involve mutating the nucleotides in question without altering the corresponding amino acids (e.g., synonymous mutations). This approach would help clarify whether the effects on readthrough are due to the nucleotide sequence, the amino acid composition, or both.

**Response:**

We appreciate the reviewer’s suggestion to further inquire whether the observed effects on TR are driven by the nucleotide sequence itself or by the resulting amino acid composition. To address this, we have designed constructs to systematically unravel these effects.

We will generate a control construct by introducing a C-to-T mutation at position -7 in the wild-type *MDH1* SCC. This mutation preserves the encoded serine, ensuring that the amino acid sequence remains unchanged. This construct will allow us to isolate the effect of the nucleotide substitution at position -7 and evaluate its potential impact on TR efficiency.

Further, we will compare this control construct (-7 T) with our previously tested *MDH1* -9,-8 mutant, which showed a strong TR increase. By introducing a C-to-T substitution at position -7 in the -9,-8 mutant, the resulting sequence (AAT) will encode the same amino acid (asparagine) as the original -9,-8 construct. This comparison will help determine whether the observed TR effects are influenced by nucleotide identity at -9,-8 or by the encoded amino acid sequence.

To specifically assess the role of the encoded amino acid, we will introduce mutations at position -7 that alter the amino acid sequence while keeping the mutated AA sequence at -9,-8 unchanged. Specifically, we will substitute position -7 with A or G, which results in different amino acids while maintaining the same nucleotide identity at -9,-8. Notably, the AAA sequence (encoding lysine) originates from LDHB, providing an additional reference for comparison.

If the substitution at position -7 that preserves the original amino acid (AAC → AAT) does not affect TR, but mutations that change the encoded amino acid (e.g., AAC → AAA or AAG) do, this would indicate that the amino acid at this position contributes to TR regulation. However, if TR efficiency remains similar across all -7 mutations while the mutated -9,-8 AA sequence is unchanged, this would reinforce the argument that the nucleotide sequence at -9,-8 is the primary determinant in the evolutionary adaptation of the high-TR motif. Taken together, we believe that this approach will provide clearer mechanistic insights into the relative contributions of nucleotides and amino acids to TR.

These experiments are summarized in the following figure:

**Reviewer #2 (Evidence, reproducibility and clarity (Required)):**
UGA is the most TR prone, however, it would be highly informative - in regard to clinical research - to test UAA and UAG stop codons too. For example, the effects of +5 and +6 on TR were shown to be stop codon dependent.

All three tested contexts (MDH1, AQP4, LDHB) are, to some degree, optimized for TR. Hence, it is expected that the whole SSC is optimized either to encode specific amino acids and/or to contain secondary structures promoting TR. For this reason, presented results might be observable only in the optimized SCCs. We think that control SSC that is not optimized for TR would represent a valuable negative control.

**Response:**

We agree with the reviewer’s suggestion to investigate UAA and UAG stop codons in addition to UGA, particularly in the context of clinical relevance. As previous studies have shown that TR efficiency can be stop codon-dependent, we agree that testing alternative stop codons could provide valuable insights.

To address this, we have designed new constructs where we replace the stop codon (UGA 🡪 UAG, and UAA) within the **`best`** and **`worst`** SCC contexts (e.g., MDH1 -9, -8). This approach will allow us to determine whether the observed SCC effects are specific to UGA or if they extend to other stop codons as well.

Furthermore, this expansion aligns with our plan to investigate SCC variations in a clinically relevant context, such as the **Rett syndrome p.R198X mutation** (Lebeda et al., 2024). We will test whether exchanging key positions (-9, -8, +11, +12) in this disease-associated SCC influences TR efficiency and whether these effects are modulated by different stop codons.

We believe this additional analysis will further strengthen the translational relevance of our findings.

Have the authors tested the +8, +11 and +12 combination? Also, could they, based on the knowledge gained, create a superpower SSC and test it in their system plus in the natural set-up? In similar manner, would it be possible to combine the best performing upstream and downstream SCCs. Do they have an additive effect?

**Response:**

We thank the reviewers for their constructive idea to test the additive effect. To address this, we have designed new constructs with the *MDH1* SCC, incorporating both the most potent upstream and down­stream exchanges that previously showed strong TR effects. This will allow us to analyze whether a “superpower SCC” can be generated by combining these elements.

Additionally, to further expand our understanding of SCC interactions, we will test these optimized SCC constructs with different stop codons (UGA 🡪 UAA, UAG) to determine whether the observed effects are stop codon-dependent. This will provide insights into whether certain SCC features enhance TR efficiency across multiple stop codon contexts or if the effects are restricted to UGA.

Discussion ("The amino acids in the nascent polypeptide chain possibly influence...."). It is very likely (documented by previous papers), that it is indeed not about the identity of the bases preceding a given stop codon, but about tRNAs (and their combinations) and the amino acids that they carry. This part of discussion refers to this phenomenon is very general terms. We would like to see a more direct discussion of what all the resulting mutations (alone or in combination) could cause/change in this light. We would suggest looking at the published 3D structures of mammalian ribosomes with nascent peptides to check what is the amino acid encoded by -9, -8, -7 positions contacting. Designing synonymous and missense mutations would then help to resolve this issue.

**Response:**

We appreciate the reviewer’s suggestion and will revise the discussion accordingly to provide a more detailed mechanistic interpretation and connect it with the results of the experiments on positions -9,-8 and -7 as explained before in the Response to Reviewer #1 (p. 2 & 3 - last comment).

LDHB is much less potent than MDH1, at least in this experimental set-up. Therefore, it is very surprising that -9 and -8 double swap dramatically increased TR in both constructs (Fig. 2B_2nd construct vs. Fig. 2E_2nd construct). A similar effect but with the stop codon downstream bases can be seen Fig. 3A/C_2nd construct vs. Fig. 3B/D_2nd construct. Naturally, one would expect to see an increase with the MDH1 bases replacing those of LDHB and vice versa, which is not the case. Why is that? Combination of tRNAs and amino acids in the case of -9 and -8? Most probably yes, considering that it works only in a double swap. Here again, we think that re-testing these in more natural set-up would be very helpful. At the very least, these data should be thoroughly discussed.

**Response:**

At this stage, the underlying mechanisms behind this effect remain an open question and are the subject of ongoing research in our group. Specifically, we are developing computational modeling approaches to predict how different SCC variations influence TR efficiency.

We will adapt the Discussion in regard to our new results.

## 3. Description of the revisions that have already been incorporated in the transferred manuscript

*Please insert a point-by-point reply describing the revisions that were already carried out and included in the transferred manuscript. If no revisions have been carried out yet, please leave this section empty.*

**Reviewer #1 (Evidence, reproducibility and clarity (Required)) – part from p. 1 & 2 – first comment:**

Notably it would be helpful to include the alignment of the three selected sequences alongside OPRL1, OPRK1, AQP4, and MAPK10. These genes also carry the same UGA CUA G motif but likely differ in surrounding nucleotides.

**Response:**

As we explained above (p. 1 & 2 – first comment) expanding the analysis to more sequences carrying the high-TR motif could provide further insights into sequence-specific differences in TR we have done sequence alignments including further candidates (*OPRL1*, *OPRK1*, *MAPK10*) and incorporated this analysis as a new figure (Supplementary Figure 2).

**Reviewer #1:**

**Minor comments:**
**P3, L3:** Be mindful that "CUA G" may be confusing unless you clearly indicate that this sequence is located downstream.

**Response:**

We now explained that this is the downstream sequence.

**P4:** The sequences shown in Figure 2A should ideally be presented in Figure 1A, so that readers can immediately see how they relate to the reporter construct.

**Response:**

We thank the reviewer for the advice and we agree that the Figure 1A should be clearer concerning the SCC. We adapted the Figure 1A.

**P5:** The phrase "in contrast the upstream..." is somewhat misleading. While the drug does show an effect (readthrough increases from 5% in untreated cells to 20% with G418), there appears to be no effect in comparison to the MDH1-TGA-MDH1sequence.

**Response:**

We clarified this in the manuscript.

**P10:** The importance of the 5′ sequence has also been reported in two previous papers (PMID: 38580646, 14736996).

**Response:**

We added the literature Bharti et al., 2024 (PMID: 38580646), Tork et al., 2004 (PMID: 14736996).

**Reviewer #2**

(Do they all have UGA as their genuine stop? Please mention it where appropriate.)

**Response:**

We agree that it is valuable to mention whether all three genes naturally terminate with UGA. We have clarified this in the revised manuscript.

**Reviewer #2 (Evidence, reproducibility and clarity (Required)):**

Unless we overlooked it, the authors should provide an excel file with all raw measurements that they used to generate the plots and perform statistical analysis.

**Response:**

We have now attached the raw data in an excel file.

The data with G418 does not seem to add any more depth to the whole story, other than the fact that: "G418 induction amplifies these effects in a manner consistent with the baseline trends", which is stated later in the text (Page 7). We think that this statement (the purpose of the G418 use) should be mentioned as soon as G418 is mentioned for the first time in the text.

**Response:**

We agree and mention the purpose of the G418 use earlier in the text.

It is not clear why OPRL1 and DUS4L were mentioned. The authors note the importance of codon in the P-site directly preceding the stop codon, however this was not investigated or discussed in this work.

**Response:**

These genes were referenced in our manuscript because the mentioned study has demonstrated that *OPRL1* exhibits high TR efficiency, whereas *DUS4L* has a low TR efficiency among human genes carrying the UGA CUA G motif. Loughran et al. systematically tested various P-site codons using a Dual-Luciferase-Assay, demonstrating that certain codons significantly modulate TR efficiency. While they provided a broad systematic analysis, our approach also shows a large impact of the altered P-site in the *MDH1* SCC (Loughran et al., 2021).

We corrected the citation and clarified this in the revised manuscript. The second reference in the citation was the correct one.

**Reviewer #2**

**Minor comments:**

What validation method(s) was used to ensure that all constructs tested were intact and stably expressed?

**Response:**

Before conducting experiments, all constructs were verified by Sanger sequencing to confirm that the intended mutations were correctly introduced and that no unintended mutations were present. We then transiently transfected the HeLa cells. To assess expression stability, we applied fluorescence intensity thresholds during data acquisition. Only cells that exhibited stable tagRFP expression above the defined threshold were included in the analysis, ensuring that only cells with robust construct expression were considered.

SD values in Figure 2B and Figure 3 seem to be of a considerable range. We suggest showing the individual measurements as dots, and perhaps increasing the number of biological or technical replicates?

Page 5, very top. Should this "increased efficiency by 4.8%." be formulated as "increased efficiency to non-significant 4.8%."?

**Response:**

Thank you for the advice. We adapted the wording.

Page 6, 2nd para. "Consequently" is not really fitting. What about "Collectivelly"?

**Response:**

We adapted the wording.

Page 13, Statistics - a typo: one-was ANOVA -> one way ANOVA

**Response:**

We corrected this.

**Reviewer #3 (Significance (Required)):
Major comments**
Overall the manuscript is well written and well designed. However, my main concern with the data presented here is that, as far as I can tell, all constructs were assayed relative to a single sense control construct. I am not sure whether fluorescent protein folding is impacted by N- or C-terminal additions, but at least for dual luciferase assays it is advisable to control for possible effects on folding by either insulating the test sequence with StopGo/2A elements, or else by generating sense controls for each test construct with amino acid sequence identical (assuming UGA is decoded as W most of the time) to the test construct. I think that fluorescent poteins tend to be more tolerant of N- and C-terminal fusions, therefore, I am not suggesting that the authors should generate many new constructs. Instead, it would be very useful to provide the fluorescent signals for RFP in particular as these values should not vary between constructs (if the extended SCC has no impact on folding).

**Response:**

We thank the reviewer for the insightful comment regarding the potential impact of N- or C-terminal fusions on fluorescent protein folding.

To address this concern, we have extracted and graphically analyzed all tagRFP fluorescence values across our experimental constructs. While some variation between constructs is visible, the tagRFP intensities of all the constructs tested are in a comparable range. There is no systematic relationship between tagRFP intensity and the effects studied.

We did not generate additional sense controls for each test construct. To ensure transparency, we will include the extracted tagRFP fluorescence data as a new figure (Supplementary Figure 3) in the revised manuscript and explicitly state this validation in the methods section.

The plots display the mean RFP intensity values for adapted constructs, derived from technical replicates. Each data point represents an individual measurement, while the bars indicate the mean and SD. The data illustrate the variability in RFP expression across different constructs, providing insight into potential influences of sequence modifications on fluorescent protein stability and expression levels.

**Reviewer #3:**

**Minor comments**
Introduction
'Eukaryotic cells universally recognize the three stop codons UAA, UAG, and UGA' this statement isn't quite true as there are many instances of reassignment of these codons to sense codons. I suggest adding 'almost' before universally.

**Response:**

Thank you for this comment, we adapted the wording.

'Crosslinking experiments have shown the interaction of eRF1 with the +4 nucleotide immediately following the stop codon [34].' I think the more direct evidence of eRF1 interacting with the +4 nt comes from the cryoEM studies (Ramakrishnan and Beckman labs).

**Response:**

Thank you for the advice. We incorporated the literature.

## 4. Description of analyses that authors prefer not to carry out

*Please include a point-by-point response explaining why some of the requested data or additional analyses might not be necessary or cannot be provided within the scope of a revision. This can be due to time or resource limitations or in case of disagreement about the necessity of such additional data given the scope of the study. Please leave empty if not applicable.*

**Reviewer #1 (Evidence, reproducibility and clarity (Required)):**

It is not clear why the authors did not test the nucleotides A(-10), A(7), C(-4), G(-1), which are specific to LDHB and not present in the other two sequences. Moreover the effect of each nucleotide is likely dependent on the broader surrounding sequence context, so testing isolated changes may not be the best approach. In some cases, these mutations also change the encoded amino acid-an amino acid that does not exist in both MDH1 and LDHB-further complicating the interpretation of the results.

**Response:**

We acknowledge the reviewer’s concern regarding the specific nucleotides A(-10), A(7), C(-4), and G(-1) in *LDHB*, which are not present in *MDH1* and *AQP4*. As described in our manuscript, ***MDH1* and *AQP4* exhibit higher basal TR efficiencies than *LDHB*.** This can be partially attributed to their shared high-TR motif (Figure 2A, highlighted in green) and an additional set of eight SCC nucleotides (Figure 2A, highlighted in blue/purple), which differ in *LDHB* (highlighted in yellow).

To investigate the contributions of these nucleotides, we systematically **exchanged individual nucleotides and nucleotide pairs** between *MDH1* and *LDHB*. However, rather than testing every possible isolated mutation, we focused on substitutions that were most likely to influence TR based on sequence conservation and previously identified positions (e.g. -9, -8, +11, +12).

We agree that an exhaustive analysis of all nucleotide positions would be valuable but also recognize that TR efficiency is influenced by the broader sequence context. Instead of testing every single position individually, we are working on a computational modeling approach to assess how different SCC variants affect readthrough efficiency. This will allow us to identify patterns and interactions between nucleotides that would not be evident from isolated mutations alone. The predictions from this model will guide future experimental studies by prioritizing nucleotide positions that have the strongest predicted impact on TR.

**Reviewer #2 (Evidence, reproducibility and clarity (Required)):**

In addition to Fig. 1B, it would be interesting to compare the efficiency of TR of the three genes of interest (Do they all have UGA as their genuine stop? Please mention it where appropriate.) in cellulo in their natural settings; i.e. without using the dual fluorescence assay. Using this more natural set-up, we would be curious to see if the authors can reproduce their results with positions -9, -8, +11 and +12. We think that the outcome of such analysis would be more physiological.

**Response:**

The aim of our study was not to measure absolute TR efficiency in physiological contexts but rather to dissect the stop codon-proximate sequence determinants that drive TR. The dual fluorescence assay allows for quantitative, high-throughput, and controlled measurements of TR efficiency across different sequence variants. The controlled experimental setup of this assay further ensures that observed differences are solely due to SCC variations rather than additional biological factors.

In one of our previous studies (Lebeda et al., 2024), we found that dual reporter measurements and Western blot TR assays with full-lengths cDNA largely align with each other, supporting the validity of this experimental system for studying TR mechanisms.

We agree with the reviewer that it would be interesting and rewarding to study TR in a more physiological setting, in order to e.g. Include effects of nonsense mediated decay (NMD). This, however, remains experimentally challenging, as it would require the tagging and stop codon manipulation on the gene level (i.e. through gene editing).
