# Supplementary material for: Defining the high-translational readthrough stop codon context
Source: PLoS Genet. 2025 Jun 25;21(6):e1011753. doi: 10.1371/journal.pgen.1011753 (PMC12233894; doi:10.1371/journal.pgen.1011753)
Supplement: Attachment — Submitted filename: Smoljanow et al_rebuttal.docx [file pgen.1011753.s007.docx]

**Manuscript number:** #RC-2024-02839

**Corresponding author(s):** Prof. Sven Thoms

Dear Editors and Reviewers,

we thank the reviewers for their constructive and helpful comments and suggestions on our manuscript titled `Defining the high-translational readthrough stop codon context`. Based on these, we have conducted new experiments and improved manuscript. Below, we provide a detailed response to each comment. We are convinced that the changes have improved the quality and clarity of the paper.

**Reviewer #1 (Evidence, reproducibility and clarity (Required)):**

Overall, this article is well written, and it addresses an interesting research question. However, there are a few methodological points that deserve further attention, as they currently limit the relevance and robustness of the work. Below are the major points I would like to highlight:

- The rationale for comparing only three sequences is unclear, given that at least seven sequences have been described with the same UGA CUA G motif. It would be more relevant and informative to compare the differences among these seven sequences rather than focusing on just three. Notably it would be helpful to include the alignment of the three selected sequences alongside OPRL1, OPRK1, AQP4, and MAPK10. These genes also carry the same UGA CUA G motif but likely differ in surrounding nucleotides. In my opinion considering all seven sequences is particularly relevant given that OPRL1 reportedly has a readthrough efficiency of 30%, much higher than the 8% reported for MDH1.

**Response:**

The rationale and the primary goal of our study were to focus on those gene products that are best characterized with regard to the functional consequences of endogenous translational readthrough. The three sequences we originally selected *(MDH1*, *AQP4*, and *LDHB*) have high levels of readthrough and well-documented functional readthrough events with established biological roles. So, our initial selection was not based on the sheer presence of the UGA_CUA_G (which can be found in > 50 transcripts) motif, but included a well-established functional relevance. We implicitly assumed that readthrough in highly expressed transcripts that have a clear functional consequence, such as the peroxisome import for LDHBx and MDH1x, have undergone a stronger evolutionary (sequence) selection than those where the high-readthrough motif is present, but expression is low and/or functional consequences of readthrough are less well established. We thank the reviewer for raising this point and we have now more carefully explained the rationale for our selection in the manuscript.

*(****Introduction****: p. 3 – last paragraph;* ***Results*** *(The influence of the 5´ context on translational readthrough efficiency surpasses that of the 3´ context beyond the CUA G motif): p. 4 – paragraph 2)*

We also agree that expanding the analysis to all seven sequences carrying the UGA_CUA_G motif could provide further insight into sequence-specific differences in translational readthrough efficiency. To address this, we have prepared sequence alignments including more candidates (*OPRL1*, *OPRK1*, *MAPK10*) and incorporated this analysis as a new panel in Figure 1 (Figure 1B). This alignment highlights conserved and divergent nucleotide positions, allowing us to contextualize our findings within a broader sequence framework.

Furthermore, we have cloned these additional sequences and incorporated them into our reporter system to evaluate their TR efficiency under our experimental conditions. These results are included in the revised manuscript (Figure 1C).

*(****Results*** *(The influence of the 5´ context on translational readthrough efficiency surpasses that of the 3´ context beyond the CUA G motif): p. 4 – paragraph 2;* ***Discussion*** *(Influence of individual nucleotides in the 5´ stop codon context and the combinatorial effect of nucleotides): p. 11 – paragraph 2).*

We reviewed the literature regarding functional extension evidence for the additional candidates *OPRL1,* *OPRK1*, and *MAPK10*. To our knowledge, there is currently no published evidence demonstrating that the readthrough extensions of these proteins are functionally characterized. Nevertheless, we included these genes in the revised manuscript as potential readthrough candidates. However, as stated above, the main focus remains on transcripts with documented functional extensions, as these provide stronger support for our conclusions on readthrough efficiency and biological relevance.

It is not clear why the authors did not test the nucleotides A(-10), A(7), C(-4), G(-1), which are specific to LDHB and not present in the other two sequences. Moreover the effect of each nucleotide is likely dependent on the broader surrounding sequence context, so testing isolated changes may not be the best approach. In some cases, these mutations also change the encoded amino acid-an amino acid that does not exist in both MDH1 and LDHB-further complicating the interpretation of the results.

**Response:**

We acknowledge the reviewer’s concern regarding the specific nucleotides A(-10), A(7), C(-4), and G(-1) in *LDHB*, which are not present in *MDH1* and *AQP4*. As described in our manuscript, ***MDH1* and *AQP4* exhibit higher basal TR efficiencies than *LDHB*.** This can be partially attributed to their shared high-TR motif (Figure 2A, highlighted in green) and an additional set of eight SCC nucleotides (Figure 2A, highlighted in blue/purple), which differ in *LDHB* (highlighted in yellow).

To investigate the contributions of these nucleotides, we systematically **exchanged individual nucleotides and nucleotide pairs** between *MDH1* and *LDHB*. However, rather than testing every possible isolated mutation, we focused on substitutions that were most likely to influence TR based on sequence conservation and previously identified positions (e.g. -9, -8, +11, +12).

We agree that an exhaustive analysis of all nucleotide positions would be valuable, but also recognize that TR efficiency is influenced by the broader sequence context. Instead of testing every single position individually, we are working on a computational modeling approach to assess how different SCC variants affect readthrough efficiency. This will allow us to identify patterns and interactions between nucleotides that would not be evident from isolated mutations alone. The predictions from this model will guide future experimental studies by prioritizing nucleotide positions that have the strongest predicted impact on TR.

I am wondering of the results are limited to the UGA CUA G motif or not? It would be valuable to explore if the same observations hold when replacing UGA CUA G with an inefficient context or with another efficient readthrough motif (e.g., CAA UUA).

**Response:**

We intended to analyze the high-readthrough motif, and UAA and UAG are not part thereof. However, we find the question raised by the reviewer interesting and important, because it could extend of findings to the modulation of more stop codon contexts that are associated with clinically relevant mutations.

To address this point, we have performed additional experiments utilizing a disease-associated premature termination codon (PTC) context, specifically the p.R198X mutation associated with Rett syndrome, as reported by Lebeda et al., 2024. This SCC has been shown to exhibit relatively high TR efficiency, but does not represent an evolutionarily adapted readthrough context. By testing the effects of -9, -8, +11, and +12 in this alternative SCC, we determined whether the identified nucleotide positions influence TR efficiency in a non-canonical TR context. This provides valuable insight into the more general context of our findings, and we thank the reviewer for raising this point

*(Figure 4D;* ***Results:*** *(Combining nucleotide positions reveals a complex interplay)* *p.9 – paragraph 2;* ***Discussion*** *(Influence of individual nucleotides in the 5´ stop codon context and the combinatorial effect of nucleotides): p.11 – paragraph 5).*

The discussion extensively addresses the importance of amino acids in the ribosome exit tunnel. To strengthen this argument, the authors should consider experiments that involve mutating the nucleotides in question without altering the corresponding amino acids (e.g., synonymous mutations). This approach would help clarify whether the effects on readthrough are due to the nucleotide sequence, the amino acid composition, or both.

**Response:**

We appreciate the reviewer’s suggestion to further inquire whether the observed effects on TR are driven by the nucleotide sequence itself or by the resulting amino acid composition.

To address the relative contributions of nucleotide sequence versus amino acid identity to translational readthrough (TR), we performed systematic mutagenesis experiments at nucleotide position -7, in addition to previously tested substitutions at positions -9 and -8.

Specifically, we generated a control construct harboring a C-to-T mutation at position -7, which preserves the encoded serine amino acid as the *MDH1* wild-type, allowing us to isolate the effect of the nucleotide change alone. This mutation resulted in an increased TR efficiency compared to the wild-type sequence.

Further, we introduced triple substitutions at positions -9, -8, and -7 encoding either asparagine (AAT, synonymous with AAC), or lysine (AAA, AAG). Despite encoding the same amino acid, these codon variants showed different TR efficiencies, suggesting that nucleotide identity rather than amino acid identity predominantly influences TR efficiency in this context.

Our results indicate that changes in nucleotide sequence at critical positions have a stronger effect on TR efficiency than the corresponding amino acid changes at position -3 of the nascent polypeptide chain. This supports the view that evolutionary conservation of the high-TR motif is primarily driven by nucleotide sequence rather than by amino acid composition.

We have now included these data and interpretations in the revised manuscript, which strengthens the mechanistic understanding of TR regulation.

*(Figure 4E;* ***Results*** *(Combining nucleotide positions reveals a complex interplay): p. 9-10 – last paragraph p. 9;* ***Discussion*** *(Codon context and sequence architecture as determinants of readthrough efficiency): p. 12 – paragraph 2)*

**Minor comments:**
**P3, L3:** Be mindful that "CUA G" may be confusing unless you clearly indicate that this sequence is located downstream.

**Response:**

We have now explained that this is the downstream sequence *(Introduction: p. 3 – paragraph 1).*

**P4:** The sequences shown in Figure 2A should ideally be presented in Figure 1A, so that readers can immediately see how they relate to the reporter construct.

**Response:**

We thank the reviewer for the advice, and we agree that Figure 1A should be clearer concerning the SCC. We adapted Figure 1A and introduced an additional panel with a sequence alignment showing the SCCs (Figure 1B). We kept Figure 2A as an overview for the specific exchanges within the sequences of *MDH1* and *LDHB*.

**P5:** The phrase "in contrast the upstream..." is somewhat misleading. While the drug does show an effect (readthrough increases from 5% in untreated cells to 20% with G418), there appears to be no effect in comparison to the MDH1-TGA-MDH1sequence.

**Response:**

We clarified this in the manuscript.

*(Results (The influence of the 5´ context on translational readthrough efficiency surpasses that of the 3´ context beyond the CUA G motif): p. 5-6 – last paragraph p.5)*

**P10:** The importance of the 5′ sequence has also been reported in two previous papers (PMID: 38580646, 14736996).

**Response:**

We added the literature Bharti et al., 2024 (PMID: 38580646), Tork et al., 2004 (PMID: 14736996.)

*(****Discussion*** *(Influence of individual nucleotides in the 5´ stop codon context and the combinatorial effect of nucleotides): p 10 – last paragraph)*

In conclusion, the manuscript presents an interesting topic and is generally well structured. Addressing the points above-particularly expanding the comparison to include all relevant sequences and clarifying the role of nucleotide context-would significantly improve the clarity and impact of this work.

**Response:**

We thank the reviewer for the positive evaluation of our work.

**Reviewer #1 (Significance (Required)):**

This work aims at determining which nucleotides are important for stop codon readthrough in humans when the UGA codon is followed by the CUAG motif. It is a classic type of study that has already been done extensively in other contexts, so it does not offer great originality in itself. However, the specific question regarding the genes involved is interesting, and the more data we gather about the role of the nucleotide context, the more useful it will be for understanding the mechanism of action. In this respect, the work is interesting. However, I consider it to be somewhat lacking in depth and believe it would benefit from a more thorough exploration of aspects indicated above.

The community interested will be the community specialized in the study of translation fidelity and stop codon readthrough.

**Reviewer #2 (Evidence, reproducibility and clarity (Required)):**

Translational readthrough (TR) has recently become the focus of many researches as its exploitation may benefit the development of pharmacological therapy for diseases caused by premature stop codon mutations. In this manuscript, Smoljanow et al. investigated the importance of a broader stop codon context (SCC) surrounding UGA stop codon followed by a high-translational readthrough (TR) sequence CUA G. Experiments were done in three model contexts, specifically MDH1, AQP4 and LDHB that were previously shown to have increased TR efficiency. By testing the single and double variations of selected SCC sequences the authors have identified positions -9, -8 and +11, +12 (U in UGA stop codon being +1) to have the most pronounced effect on the TR in their high TR setup. While the effect of SCC on TR has been investigated before, the impact of identified positions on TR in mammalian cells represents a novel information highly relevant to basic and clinical research.

To explore the effect of SCC on the TR, the authors have employed dual fluorescence reporter system consisting of tagRFP and eGFP genes separated by different TR motives containing UGA stop codon. The fluorescence was measured in HeLa cells in three biological replicates and the results are presented as bar charts with indicated SD values and significance.

**Major comments:**
UGA is the most TR prone, however, it would be highly informative - in regard to clinical research - to test UAA and UAG stop codons too. For example, the effects of +5 and +6 on TR were shown to be stop codon dependent.

All three tested contexts (MDH1, AQP4, LDHB) are, to some degree, optimized for TR. Hence, it is expected that the whole SSC is optimized either to encode specific amino acids and/or to contain secondary structures promoting TR. For this reason, presented results might be observable only in the optimized SCCs. We think that control SSC that is not optimized for TR would represent a valuable negative control.

**Response:**

We appreciate the reviewer’s suggestion to investigate UAA and UAG stop codons in addition to UGA, particularly in the context of clinical relevance. As previous studies have shown that TR efficiency can be stop codon-dependent, we agree that testing alternative stop codons could provide valuable insights.

To address this, we designed new constructs where we replaced the stop codon (UGA 🡪 UAG, and UAA) within the **`best`** and **`worst`** SCC contexts (e.g., MDH1 -9, -8). This approach allowed us to determine whether the observed SCC effects are specific to UGA or if they extend to other stop codons as well.

*(Figure 4A-C;* ***Results:*** *(Combining nucleotide positions reveals a complex interplay)* *p.9 – paragraph 1-2;* ***Discussion*** *(Influence of individual nucleotides in the 5´ stop codon context and the combinatorial effect of nucleotides): p.11 – paragraph 3-4)*

Furthermore, this expansion aligned with our plan to investigate SCC variations in a clinically relevant context, such as the **Rett syndrome p.R198X mutation** (Lebeda et al., 2024). We tested whether exchanging key positions (-9, -8, +11, +12) in this disease-associated SCC influences TR efficiency and whether these effects are modulated by different stop codons (Figure 4D).

***Results:*** *(Combining nucleotide positions reveals a complex interplay)* *p.9 – paragraph 2;* ***Discussion*** *(Influence of individual nucleotides in the 5´ stop codon context and the combinatorial effect of nucleotides): p.11 – paragraph 5).*

In addition to Fig. 1B, it would be interesting to compare the efficiency of TR of the three genes of interest (Do they all have UGA as their genuine stop? Please mention it where appropriate.) in cellulo in their natural settings; i.e. without using the dual fluorescence assay. Using this more natural set-up, we would be curious to see if the authors can reproduce their results with positions -9, -8, +11 and +12. We think that the outcome of such analysis would be more physiological.

**Response:**

The aim of our study was not to measure absolute TR efficiency in physiological contexts but rather to dissect the stop codon-proximate sequence determinants that drive TR. The dual fluorescence assay allows for quantitative, high-throughput, and controlled measurements of TR efficiency across different sequence variants. The controlled experimental setup of this assay further ensures that observed differences are solely due to SCC variations rather than additional biological factors.

In one of our previous studies (Lebeda et al., 2024), we found that dual reporter measurements and Western blot TR assays with full-lengths cDNA largely align with each other, supporting the validity of this experimental system for studying TR mechanisms.

We agree with the reviewer that it would be interesting and rewarding to study TR in a more physiological setting, in order to e.g. Include effects of nonsense mediated decay (NMD). This, however, remains experimentally more demanding than using cDNA, as it would require the tagging and stop codon manipulation on the gene level (i.e. through gene editing).

Unless we overlooked it, the authors should provide an excel file with all raw measurements that they used to generate the plots and perform statistical analysis.

**Response:**

We attached the raw data in a supplementary Excel file.

The data with G418 does not seem to add any more depth to the whole story, other than the fact that: "G418 induction amplifies these effects in a manner consistent with the baseline trends", which is stated later in the text (Page 7). We think that this statement (the purpose of the G418 use) should be mentioned as soon as G418 is mentioned for the first time in the text.

**Response:**

We mentioned the purpose of the G418 use earlier in the text.

*(Introduction: p.5 – last paragraph)*

LDHB is much less potent than MDH1, at least in this experimental set-up. Therefore, it is very surprising that -9 and -8 double swap dramatically increased TR in both constructs (Fig. 2B_2nd construct vs. Fig. 2E_2nd construct). A similar effect but with the stop codon downstream bases can be seen Fig. 3A/C_2nd construct vs. Fig. 3B/D_2nd construct. Naturally, one would expect to see an increase with the MDH1 bases replacing those of LDHB and vice versa, which is not the case. Why is that? Combination of tRNAs and amino acids in the case of -9 and -8? Most probably yes, considering that it works only in a double swap. Here again, we think that re-testing these in more natural set-up would be very helpful. At the very least, these data should be thoroughly discussed.

**Response:**

At this stage, the underlying mechanisms behind this effect remain an open question and are the subject of ongoing research in our group. Specifically, we are developing computational modeling approaches to predict how different SCC variations influence TR efficiency. We adapted the Discussion regarding our new results.

Have the authors tested the +8, +11 and +12 combination? Also, could they, based on the knowledge gained, create a superpower SSC and test it in their system plus in the natural set-up? In similar manner, would it be possible to combine the best performing upstream and downstream SCCs. Do they have an additive effect?

**Response:**

We thank the reviewers for their constructive idea to test the additive effect. In response, we generated new constructs based on the *MDH1* SCC, incorporating both the most potent upstream and downstream exchanges that previously showed strong TR effects. This approach allowed us to test whether a “superpower SCC” can be generated by combining these elements.

Additionally, to further expand our understanding of SCC interactions, we tested these optimized SCC constructs with different stop codons (UGA 🡪 UAA, UAG). This allowed us to determine whether the observed effects are stop codon-dependent. This provided insights into whether certain SCC features enhance TR efficiency across multiple stop codon contexts or if the effects are restricted to UGA. We added a new Figure for this data (Figure 4A-C).

*(****Results:*** *(Combining nucleotide positions reveals a complex interplay)* *p.9 – paragraph 1-2;* ***Discussion*** *(Influence of individual nucleotides in the 5´ stop codon context and the combinatorial effect of nucleotides): p.11 – paragraph 3-4).*

Discussion ("The amino acids in the nascent polypeptide chain possibly influence...."). It is very likely (documented by previous papers), that it is indeed not about the identity of the bases preceding a given stop codon, but about tRNAs (and their combinations) and the amino acids that they carry. This part of discussion refers to this phenomenon is very general terms. We would like to see a more direct discussion of what all the resulting mutations (alone or in combination) could cause/change in this light. We would suggest looking at the published 3D structures of mammalian ribosomes with nascent peptides to check what is the amino acid encoded by -9, -8, -7 positions contacting. Designing synonymous and missense mutations would then help to resolve this issue.

**Response:**

We appreciate the reviewer’s suggestion and raised the discussion accordingly and provide a more detailed mechanistic interpretation and connected it with the results of the experiments on positions -9,-8 and -7 as explained before in the Response to Reviewer #1 (p. 3 - comment Reviewer #1 (Evidence, reproducibility and clarity (Required) - last major point) (Figure 4E).

*(****Results*** *(Combining nucleotide positions reveals a complex interplay): p. 9-10 – last paragraph p. 9;* ***Discussion*** *(Codon context and sequence architecture as determinants of readthrough efficiency): p. 12 – paragraph 2).*

It is not clear why OPRL1 and DUS4L were mentioned. The authors note the importance of codon in the P-site directly preceding the stop codon, however this was not investigated or discussed in this work.

**Response:**

These genes were referenced in our manuscript because the mentioned study has demonstrated that *OPRL1* exhibits high TR efficiency, whereas *DUS4L* has a low TR efficiency among human genes carrying the UGA CUA G motif. Loughran et al. systematically tested various P-site codons using a Dual-Luciferase-Assay, demonstrating that certain codons significantly modulate TR efficiency. While they provided a broad systematic analysis, our approach also shows a large impact of the altered P-site in the *MDH1* SCC (cf. Loughran et al., 2021). We corrected the citation and clarified this in the revised manuscript. The second reference in the citation was the correct one.

**Minor comments:**

What validation method(s) was used to ensure that all constructs tested were intact and stably expressed?

**Response:**

Before conducting experiments, all constructs were verified by Sanger sequencing to confirm that the intended mutations were correctly introduced and that no unintended mutations were present. We then transiently transfected the HeLa cells. To assess expression stability, we applied fluorescence intensity thresholds during data acquisition. Only cells that exhibited stable tagRFP expression above the defined threshold were included in the analysis, ensuring that only cells with robust construct expression were considered.

SD values in Figure 2B and Figure 3 seem to be of a considerable range. We suggest showing the individual measurements as dots, and perhaps increasing the number of biological or technical replicates?

Page 5, very top. Should this "increased efficiency by 4.8%." be formulated as "increased efficiency to non-significant 4.8%."?

**Response:**

Thank you for the advice. We adapted the wording.

Page 6, 2nd para. "Consequently" is not really fitting. What about "Collectivelly"?

**Response:**

We adapted the wording.

Page 13, Statistics - a typo: one-was ANOVA -> one way ANOVA

**Response:**

We corrected this.

We thank you for the opportunity to review this article.
Leos Shivaya Valasek & Filip Brázdovič

Reviewer #2 (Significance (Required)):

This study logically expands previous research from Dr. Thoms lab using the well-established tools and well-studied genes. It is technically sound, well written, however, in our opinion only half done. The conclusions are interesting but also "surprising" in a way that is not easily explained, and more work should be invested into this project to address this important phenomenon in greater detail, which could lead to more logical and applicable conclusions. More details above.

Current advance is considered incremental, but with more data it may become functional/important.

Audience will consist of basic researchers interested in translation control, as well as in translational/clinical research.

**Reviewer #3 (Evidence, reproducibility and clarity (Required)):**

Smoljanow et al explore the wider readthrough context of the leaky UGA_CUAG motif by examining in greater detail the signals surrounding MDH1, AQP4 and LDHB. Here they use a fused dual fluorescence reporter system where readthrough events result in translation of an RFP-GFP fusion protein whereas termination at the tested stop codon results in synthesis of RFP. To gain an understanding of the influence of sequences 5' and 3' of the UGA_CUAG, Smoljanow et al generated cross-combined and single or double nucleotide mutant constructs. Reporter constructs were transfected into HeLa cells and readthrough efficiencies were calculated by determining the RFP/GFP ratio of test constructs relative to an RFP-GFP sense control (serving as 100% readthrough). Transfectants were either left untreated or else treated with the readthrough-inducing G418.

The major conclusion drawn from these experiments is that upstream nucleotide positions -9 and -8 and downstream nucleotide positions +11 and +12 can influence readthrough levels.

**Reviewer #3 (Significance (Required)):
Major comments**
Overall the manuscript is well written and well designed. However, my main concern with the data presented here is that, as far as I can tell, all constructs were assayed relative to a single sense control construct. I am not sure whether fluorescent protein folding is impacted by N- or C-terminal additions, but at least for dual luciferase assays it is advisable to control for possible effects on folding by either insulating the test sequence with StopGo/2A elements, or else by generating sense controls for each test construct with amino acid sequence identical (assuming UGA is decoded as W most of the time) to the test construct. I think that fluorescent poteins tend to be more tolerant of N- and C-terminal fusions, therefore, I am not suggesting that the authors should generate many new constructs. Instead, it would be very useful to provide the fluorescent signals for RFP in particular as these values should not vary between constructs (if the extended SCC has no impact on folding).

**Response:**

We thank the reviewer for the insightful comment regarding the potential impact of N- or C-terminal fusions on fluorescent protein folding.

To address this concern, we extracted and analyzed all tagRFP fluorescence values across our experimental constructs. While some variation between constructs is visible, the tagRFP intensities of all the constructs tested showed a comparable range. There is no systematic relationship between tagRFP intensity and the effects studied. For transparency, we included the extracted tagRFP fluorescence data as a new figure (Supplementary Figure 2A). However, we did not generate additional sense controls for each test construct.

**Minor comments**
Introduction
'Eukaryotic cells universally recognize the three stop codons UAA, UAG, and UGA' this statement isn't quite true as there are many instances of reassignment of these codons to sense codons. I suggest adding 'almost' before universally.

**Response:**

Thank you for this comment, we adapted the wording.

'Crosslinking experiments have shown the interaction of eRF1 with the +4 nucleotide immediately following the stop codon [34].' I think the more direct evidence of eRF1 interacting with the +4 nt comes from the cryoEM studies (Ramakrishnan and Beckman labs).

**Response:**

Thank you for the advice. We incorporated the literature.

**Significance**

Although a similar study testing different UGA_CUAG contexts was recently published (ref 43), it is still important to ascertain the details of those cis-acting sequences that impact termination efficiency on other motifs. These findings will be useful for any researchers interested in translation termination and stop codon readthrough.
