# Supplementary material for: Defining the high-translational readthrough stop codon context
Source: PLoS Genet. 2025 Jun 25;21(6):e1011753. doi: 10.1371/journal.pgen.1011753 (PMC12233894; doi:10.1371/journal.pgen.1011753)
Supplement: Peer review history

# Editor report: Decision Letter 0

**DOI:** 10.1371/journal.pgen.1011753.r001

**Authors:** Monica P. Colaiácovo

PGENETICS-D-25-00291

Defining the high-translational readthrough stop codon context

PLOS Genetics

Dear Dr. Thoms,

Thank you for submitting your manuscript to PLOS Genetics. After careful consideration, we feel that it has merit but does not fully meet PLOS Genetics's publication criteria as it currently stands. Therefore, we invite you to proceed with the experiments you outlined in your revision plan and submit a revised version of the manuscript that addresses the points raised during the review process.

Please submit your revised manuscript within 60 days May 25 2025 11:59PM. If you will need more time than this to complete your revisions, please reply to this message or contact the journal office at plosgenetics@plos.org. When you're ready to submit your revision, log on to https://www.editorialmanager.com/pgenetics/ and select the 'Submissions Needing Revision' folder to locate your manuscript file.

Please include the following items when submitting your revised manuscript:

* A rebuttal letter that responds to each point raised by the editor and reviewer(s). You should upload this letter as a separate file labeled 'Response to Reviewers'. This file does not need to include responses to any formatting updates and technical items listed in the 'Journal Requirements' section below.

* A marked-up copy of your manuscript that highlights changes made to the original version. You should upload this as a separate file labeled 'Revised Manuscript with Track Changes'.

* An unmarked version of your revised paper without tracked changes. You should upload this as a separate file labeled 'Manuscript'.

If you would like to make changes to your financial disclosure, competing interests statement, or data availability statement, please make these updates within the submission form at the time of resubmission. Guidelines for resubmitting your figure files are available below the reviewer comments at the end of this letter.

We look forward to receiving your revised manuscript.

Kind regards,

Monica P. Colaiácovo

Section Editor

PLOS Genetics

Aimée Dudley

Editor-in-Chief

PLOS Genetics

Anne Goriely

Editor-in-Chief

PLOS Genetics

**Journal Requirements:**

1) Please provide an Author Summary. This should appear in your manuscript between the Abstract (if applicable) and the Introduction, and should be 150-200 words long. The aim should be to make your findings accessible to a wide audience that includes both scientists and non-scientists. Sample summaries can be found on our website under Submission Guidelines:

https://journals.plos.org/plosgenetics/s/submission-guidelines#loc-parts-of-a-submission

2) Please upload all main figures as separate Figure files in .tif or .eps format. For more information about how to convert and format your figure files please see our guidelines: 

https://journals.plos.org/plosgenetics/s/figures

3) We have noticed that you have uploaded Supporting Information files, but you have not included a complete list of legends. Please add a full list of legends for your Supporting Information files after the references list.

4) We notice that your supplementary Figures, and Tables are included in the manuscript file. Please remove them and upload them with the file type 'Supporting Information'. Please ensure that each Supporting Information file has a legend listed in the manuscript after the references list.

5) Please amend your detailed Financial Disclosure statement. This is published with the article. It must therefore be completed in full sentences and contain the exact wording you wish to be published.

1) State the initials, alongside each funding source, of each author to receive each grant. For example: "This work was supported by the National Institutes of Health (####### to AM; ###### to CJ) and the National Science Foundation (###### to AM)."

2) State what role the funders took in the study. If the funders had no role in your study, please state: "The funders had no role in study design, data collection and analysis, decision to publish, or preparation of the manuscript."

3) If any authors received a salary from any of your funders, please state which authors and which funders.

[NOTE: If reviewer comments were submitted as an attachment file, they will be attached to this email and accessible via the submission site. Please log into your account, locate the manuscript record, and check for the action link "View Attachments". If this link does not appear, there are no attachment files.]

**Figure resubmission:**

While revising your submission, please upload your figure files to the Preflight Analysis and Conversion Engine (PACE) digital diagnostic tool, https://pacev2.apexcovantage.com/. PACE helps ensure that figures meet PLOS requirements. To use PACE, you must first register as a user. Registration is free. Then, login and navigate to the UPLOAD tab, where you will find detailed instructions on how to use the tool. If you encounter any issues or have any questions when using PACE, please email PLOS at figures@plos.org. Please note that Supporting Information files do not need this step. If there are other versions of figure files still present in your submission file inventory at resubmission, please replace them with the PACE-processed versions.

**Reproducibility:**

To enhance the reproducibility of your results, we recommend that authors of applicable studies deposit laboratory protocols in protocols.io, where a protocol can be assigned its own identifier (DOI) such that it can be cited independently in the future. Additionally, PLOS ONE offers an option to publish peer-reviewed clinical study protocols. Read more information on sharing protocols at https://plos.org/protocols?utm_medium=editorial-email&utm_source=authorletters&utm_campaign=protocols

Attachment Submitted filename: 153738_1_rebuttal_3303107_ss1d1r.docx

# Editor report: Decision Letter 1

**DOI:** 10.1371/journal.pgen.1011753.r002

**Authors:** Monica P. Colaiácovo

Dear Dr Thoms,

We are pleased to inform you that your manuscript entitled "Defining the high-translational readthrough stop codon context" has been editorially accepted for publication in PLOS Genetics. Congratulations!

Before your submission can be formally accepted and sent to production you will need to complete our formatting changes, which you will receive in a follow up email. Please be aware that it may take several days for you to receive this email; during this time no action is required by you. Please note: the accept date on your published article will reflect the date of this provisional acceptance, but your manuscript will not be scheduled for publication until the required changes have been made.

Once your paper is formally accepted, an uncorrected proof of your manuscript will be published online ahead of the final version, unless you’ve already opted out via the online submission form. If, for any reason, you do not want an earlier version of your manuscript published online or are unsure if you have already indicated as such, please let the journal staff know immediately at plosgenetics@plos.org.

In the meantime, please log into Editorial Manager at https://www.editorialmanager.com/pgenetics/, click the "Update My Information" link at the top of the page, and update your user information to ensure an efficient production and billing process. Note that PLOS requires an ORCID iD for all corresponding authors. Therefore, please ensure that you have an ORCID iD and that it is validated in Editorial Manager. To do this, go to ‘Update my Information’ (in the upper left-hand corner of the main menu), and click on the Fetch/Validate link next to the ORCID field.  This will take you to the ORCID site and allow you to create a new iD or authenticate a pre-existing iD in Editorial Manager.

If you have a press-related query, or would like to know about making your underlying data available (as you will be aware, this is required for publication), please see the end of this email. If your institution or institutions have a press office, please notify them about your upcoming article at this point, to enable them to help maximise its impact. Inform journal staff as soon as possible if you are preparing a press release for your article and need a publication date.

Thank you again for supporting open-access publishing; we are looking forward to publishing your work in PLOS Genetics!

Yours sincerely,

Monica Colaiácovo

Section Editor

PLOS Genetics

Aimée Dudley

Editor-in-Chief

PLOS Genetics

Anne Goriely

Editor-in-Chief

PLOS Genetics

www.plosgenetics.org

Twitter: @PLOSGenetics

----------------------------------------------------

Comments from the reviewers (if applicable):

----------------------------------------------------

**Data Deposition**

If you have submitted a Research Article or Front Matter that has associated data that are not suitable for deposition in a subject-specific public repository (such as GenBank or ArrayExpress), one way to make that data available is to deposit it in the Dryad Digital Repository . As you may recall, we ask all authors to agree to make data available; this is one way to achieve that. A full list of recommended repositories can be found on our website .

The following link will take you to the Dryad record for your article, so you won't have to re‐enter its bibliographic information, and can upload your files directly: 

http://datadryad.org/submit?journalID=pgenetics&manu=PGENETICS-D-25-00291R1

More information about depositing data in Dryad is available at http://www.datadryad.org/depositing. If you experience any difficulties in submitting your data, please contact help@datadryad.org for support.

Additionally, please be aware that our data availability policy  requires that all numerical data underlying display items are included with the submission, and you will need to provide this before we can formally accept your manuscript, if not already present.

----------------------------------------------------

**Press Queries**

If you or your institution will be preparing press materials for this manuscript, or if you need to know your paper's publication date for media purposes, please inform the journal staff as soon as possible so that your submission can be scheduled accordingly. Your manuscript will remain under a strict press embargo until the publication date and time. This means an early version of your manuscript will not be published ahead of your final version. PLOS Genetics may also choose to issue a press release for your article. If there's anything the journal should know or you'd like more information, please get in touch via plosgenetics@plos.org .

Attachment Submitted filename: Smoljanow et al_rebuttal.docx

# Editor report: Acceptance letter

**DOI:** 10.1371/journal.pgen.1011753.r003

**Authors:** Monica P. Colaiácovo

PGENETICS-D-25-00291R1

Defining the high-translational readthrough stop codon context

Dear Dr Thoms,

We are pleased to inform you that your manuscript entitled "Defining the high-translational readthrough stop codon context" has been formally accepted for publication in PLOS Genetics! Your manuscript is now with our production department and you will be notified of the publication date in due course.

The corresponding author will soon be receiving a typeset proof for review, to ensure errors have not been introduced during production. Please review the PDF proof of your manuscript carefully, as this is the last chance to correct any errors. Please note that major changes, or those which affect the scientific understanding of the work, will likely cause delays to the publication date of your manuscript.

Soon after your final files are uploaded, unless you have opted out or your manuscript is a front-matter piece, the early version of your manuscript will be published online. The date of the early version will be your article's publication date. The final article will be published to the same URL, and all versions of the paper will be accessible to readers.

Thank you again for supporting PLOS Genetics and open-access publishing. We are looking forward to publishing your work!

With kind regards,

Olena Szabo

PLOS Genetics

On behalf of:

The PLOS Genetics Team

Carlyle House, Carlyle Road, Cambridge CB4 3DN | United Kingdom

plosgenetics@plos.org | +44 (0) 1223-442823

plosgenetics.org | Twitter: @PLOSGenetics
